# Transcriptome Analysis of Skin Photoaging in Chinese Females Reveals the Involvement of Skin Homeostasis and Metabolic Changes

**DOI:** 10.1371/journal.pone.0061946

**Published:** 2013-04-24

**Authors:** Wei Yan, Li-Li Zhang, Li Yan, Feng Zhang, Ning-Bei Yin, Hong-Bin Lin, Chen-Yu Huang, Lei Wang, Jun Yu, Duen-Mei Wang, Zhen-Min Zhao

**Affiliations:** 1 Plastic Surgery Hospital, Chinese Academy of Medical Sciences and Peking Union Medical College, Beijing, China; 2 Chinese Academy of Sciences Key Laboratory of Genome Sciences and Information, Beijing Institute of Genomics, Chinese Academy of Sciences, Beijing, China; 3 University of Chinese Academy of Sciences, Beijing, China; 4 China Meitan General Hospital, Beijing, China; 5 People’s Hospital of Jincheng City, Jincheng, Shanxi, China; University of Tennessee, United States of America

## Abstract

**Background:**

Photoaging is cumulative damage to skin, caused by chronic, repeated solar radiation exposure. Its molecular mechanisms are poorly understood at the level of global gene expression.

**Objective:**

This study set out to uncover genes and functional modules involved in photoaging at the level of transcription, with the use of skin samples from Chinese women.

**Methods:**

Using the Illumina microarray platform, we compared the genome-wide expression profiles of 21 pairs of sun-exposed pre-auricular and sun-protected post-auricular skin samples from northern Chinese women.

**Results:**

With microarray analysis, 1,621 significantly regulated genes due to photoaging were identified from skin samples. These genes were subjected to functional enrichment analyses with both the Gene Ontology (GO) and Kyoto Encyclopedia of Genes and Genomes (KEGG) annotation databases. As revealed by the functional analyses, the up-regulated functional modules in sun-exposed pre-auricular skin were related to various cellular activities in regulation of the skin homeostasis (e.g., the KEGG pathways *TGF-beta signaling pathway* and *ECM-receptor interaction*), whereas the down-regulated functional modules were mostly metabolic-related. Additionally, five selected genes (*HOXA5, LEPR, CLDN5, LAMC3*, and *CGA*) identified as differentially-expressed were further confirmed by quantitative real-time PCR (Q-RT-PCR).

**Conclusion:**

Our findings suggest that disruption of skin homeostasis and down-regulation of skin metabolism may play important roles in the process of photoaging.

## Introduction

Human skin undergoes both intrinsic and extrinsic aging processes [1]. Extrinsic aging is mainly caused by solar irradiation, and is termed “photoaging”. Different from acute sun damage to skin, photoaging is a cumulative process that is caused by chronic, repeated exposure to solar radiation. Photoaged skin can be characterized by a leathery appearance with wrinkles, laxity, and dyspigmentation [2]. It has been suggested that photoaging is a predominant factor in the prematurely aged appearance of sun-exposed skin [3].

Various molecules, such as collagen [4], matrix metalloproteinases [5], [6], elastin binding protein, versican [7], hyaluronic acid [8], and keratins [9], were identified as affecting the appearance of photoaged skin. In addition, at least two signaling pathways, transforming growth factor (TGF-β) signaling [10] and lipid synthesis [11], [12], were reported to be responsible for the photoaging process of skin.

For years, global gene expression profiling has been widely used in biological studies. However, to our knowledge, only two such studies reported on photoaging. The first study, performed by Urschitz *et al.*, used serial analysis of gene expression (SAGE) to compare differential expression between sun-exposed pre-auricular skin and sun-protected post-auricular skin from a 55-year-old white female [9]. Of the total 6,598 unique genes examined, they identified 25 differentially expressed genes (DEGs). The second study, performed by Robinson *et al.*, used a microarray approach to examine the expression of 20,000 plus genes from extensor-forearm skins of young vs. old age groups [12]. In their study, photoaging-related biological progresses were identified by Gene Ontology (GO) enrichment analysis. Both studies found various biological processes for photoaging, including cell growth, proliferation, differentiation and apoptosis [9], lipid biosynthesis [12], immune and inflammatory responses [9], [12], etc.

It is known that ethnic groups with different skin tones respond to solar radiation differently, for skin pigment provides a significant degree of protection against UV damage [13]. Asian populations, which tend to have a darker skin tones than that of Europeans, have not been well studied in terms of photoaging at a global gene expression level. In this study, we used a whole genome microarray platform to examine the genes and functional modules involved in the process of photoaging in Chinese women. Results derived from this study can provide insights into the mechanism of photoaging and intervention of the photoaging process.

## Materials and Methods

### Skin Samples and RNA Isolation

Skin samples were obtained from 21 healthy Chinese females (ages ranging from 34 to 55 years, with a mean age of 46.2±5.8) undergoing rhytidectomy surgery at the Plastic Surgery Hospital of Chinese Academy of Medical Sciences and Peking Union Medical College, Beijing, China (Table 1). Intact layer of skin from the front of the tragus of the ear (pre-auricular skin), which subjected to decades of natural sun-exposure was used as sun-exposed skin; skin from behind the auricle of the ear (post-auricular skin), which was UV-protected by auricle was used as sun-protected skin. The differentiation of irradiation dosages between our two groups (sun-exposed and sun-protected) were self-compared by using pre-auricular and post-auricular skin samples from the same subject. Also, all the subjects shared similar climates, occupations, skin pigment levels, and photodamage grades. All the enrolled subjects for this solar irradiation study lived in northern China, had brown skin, had indoor occupations with an average of 1–2 hours sun-exposure per day, experienced several decades (46.2±5.8 years) of repeated sun exposure, and were clinically classified as type II photodamage according to Glogau’s photoaging classification [1]. After obtaining from the rhytidectomy surgery, skin samples were immediately washed with sterile normal saline, trimmed off fat tissues, and cut into the size of 0.5×0.5 cm. The processed skin samples were snap-frozen in liquid nitrogen and stored at −80°C until RNA isolation. This study followed the Declaration of Helsinki protocols. All of the subjects used in the study provided their written informed consent. The study was approved by the Ethics Committee of the Plastic Surgery Hospital, Chinese Academy of Medical Sciences and Peking Union Medical College.

**Table 1:** Sample demographics.

Subject_ID	Sample_ID*	Age	BeadChip version#
1	1A, 1B	40	V2
2	2A, 2B	38	V2
3	3A, 3B	40	V2
4	4A, 4B	47	V2
5	5A, 5B	50	V2
6	6A, 6B	50	V2
7	7A, 7B	55	V2
8	8A, 8B	53	V2
9	9A, 9B	55	V2
10	10A, 10B	46	V3
11	11A, 11B	46	V3
12	12A, 12B	50	V3
13	13A, 13B	34	V3
14	14A, 14B	38	V3
15	15A, 15B	42	V3
16	16A, 16B	44	V3
17	17A, 17B	44	V3
18	18A, 18B	46	V3
19	19A, 19B	52	V3
20	20A, 20B	47	V3
21	21A, 21B	53	V3

* A, pre-auricular skin sample; B, post-auricular skin sample.

# V2, Illumina Human-6 V2 Expression BeadChip; V3, Illumina Human-6 V3 Expression BeadChip.

Total RNA from skin samples was isolated with the TRIzol^®^ reagent following the manufacturer’s suggestions (Invitrogen, Carlsbad, CA, USA). RNA integrity and purity were checked by gel electrophoresis and spectrophotometry, respectively. Qualified RNA samples were without noticeable degradation and were stored at −80°C prior to analysis.

### Microarray Processing

Two versions of Illumina HUMANWG-6, v2.0 and v3.0 BeadChips (Illumina, San Diego, CA, USA) were used to generate the global gene expression profiles for 9 and 12 pairs of samples, respectively (Table 1). An update of the Illumina microarray BeadChips occurred during the period of our sample collection, which led to the use of two versions of BeadChips. Regardless of the chip versions, all RNA samples were processed and analyzed with the same procedure. In brief, 500 ng of total RNA per sample was used to generate biotinylated cRNA by *in vitro* transcription with the Illumina^®^ TotalPrep Amplification Kit (Ambion, Austin, TX, USA), according to the manufacturer’s suggestions. Then, 1,500 ng of biotinylated cRNA per sample was hybridized to BeadChips. The hybridization, washing, and drying of the BeadChips were processed according to the standardized procedures from Illumina. The BeadChips were scanned with an Illumina BeadArray Reader.

### Microarray Data Analysis

The quality of the microarray data was evaluated as suggested by the Illumina microarray platform. All the detected parameters fell within their expected ranges. Raw data from HUMANWG-6 v2.0 and v3.0 BeadChips was exported using the Illumina BeadStudio v2.0 and Illumina GenomeStudio software, respectively. Only the common probes of the two versions of the BeadChip were used for the subsequent analyses. They were extracted by the R software package, based on their identical probe sequences between the two versions of BeadChip.

To determine the DEGs between sun-exposed pre-auricular and sun-protected post-auricular skin samples, GeneSpring GX 10.0 (Aglient, Santa Clara, CA, USA) software was used. First, the data was preprocessed using the default parameters and then normalized by a *quantile* algorithm for its best performance in our test study (Figure S1). Second, the expressed probes were defined as detecting over 60% of samples from either group with significant p-values (< 0.05). Third, the DEGs were obtained from the expressed probes by an *unpaired T-test* with a cutoff of *Asymptotic* p-value < 0.05. A heatmap, using the Cluster and TreeView (version 3.0) software programs, was generated with the top 100 shared DEGs between GeneSpring and Gene Set Enrichment Analysis (GSEA) [14]. The microarray data were deposited into the NCBI’s Gene Expression Omnibus (GEO) database (Series GSE38308).

### Functional Enrichment Analyses

To conduct the functional enrichment analyses, three software packages, Web-based Gene Set Analysis Toolkit (WebGestalt) [15], Database for Annotation, Visualization and Integration Discovery (DAVID) [16], and GSEA, were separately employed. Meanwhile, functional enrichment analyses were mainly focused on the two popular annotation databases: Gene Ontology (GO) and Kyoto Encyclopedia of Genes and Genomes (KEGG). The dataset for WebGestalt and DAVID was our list of DEGs, whereas the dataset for GSEA was the normalized intensities of the 37,352 common probes between the two versions of BeadChips. In WebGestalt, *WEBGESTALT-HUMAN* was used as the reference dataset, the minimum number of genes for enrichment was set at 5, and the significance analysis was performed using the *Hypergeometric test* with the significance level set at p<0.01. In DAVID, the Human genome was used as the reference; the rest of the parameters were set to their default values. In GSEA, all parameters were set to their defaults. The common enriched terms of these three software packages were used for subsequent analysis. The publication images of enriched pathways were produced with Adobe Photoshop CS3.

### Quantitative Real-time PCR

Five genes [homeobox A5 (*HOXA5*); leptin receptor (*LEPR*); claudin 5 (*CLDN5*); laminin, gamma 3 (*LAMC3*); glycoprotein hormones, alpha polypeptide (*CGA*)] selected from our DEG list were further validated by SYBR-green-based quantitative real-time PCR (Q-RT-PCR). The RNA samples used for the PCR reactions were the same as those for the microarray experiments. The housekeeping gene, *ACTB*, was used as an internal control. For each sample, 1,000 ng of total RNA was reverse transcribed into cDNA with M-MuLV Reverse Transcriptase (NEB, Ipswich, MA, USA), according to the manufacturer’s suggestions. The Q-RT-PCR reactions were performed in triplicate using a DNA Engine OpticonTM 2 thermal cycler (MJ Research, Waltham, MA, USA). Fold changes were calculated by Livak’s 2^−ΔΔCT^ method [17]. Sequences of primers and reaction/cycling conditions are provided in Table 2.

**Table 2:** Primer sets and reaction conditions of Q-RT-PCR experiments.

Gene	Primer sequences (Forward, Reverse)	Cycles	Annealing Tm (°C)	Product size (bp)
*CGA*	F: CCACTCCACTAAGGTCCAAGA	40	58	146
	R: AGTACTGCAGTGGCACG			
*LAMC3*	F: CGGGAATCGCGTATCTC	40	58	122
	R: AGTGCCCACTGAGTCTCGTTC			
*CLDN5*	F: ACGGGAGGCGTGCTCTACCTG	40	56	102
	R: GGGCACAGACGGGTCGTAAA			
*LEPR*	F: GCAAGCACATACTGTTACGGT	40	52	118
	R: AGCACTGAGTGACTGCACGAT			
*HOXA5*	F: TACCCCTGGATGCGCAAGCTG	40	58	139
	R: TGCGGGTCAGGTAACGGTTGA			
*ACTB*	F: GCAAAGACCTGTACGCCAACA	40	X*	150
	R:ACACGGAGTACTTGCGCTCAG			

* X indicates that the annealing Tm of the gene *ACTB* could be 52, 56 or 58, according to the target genes.

## Results

### Identification of Differentially Expressed Genes

**Figure 1:**
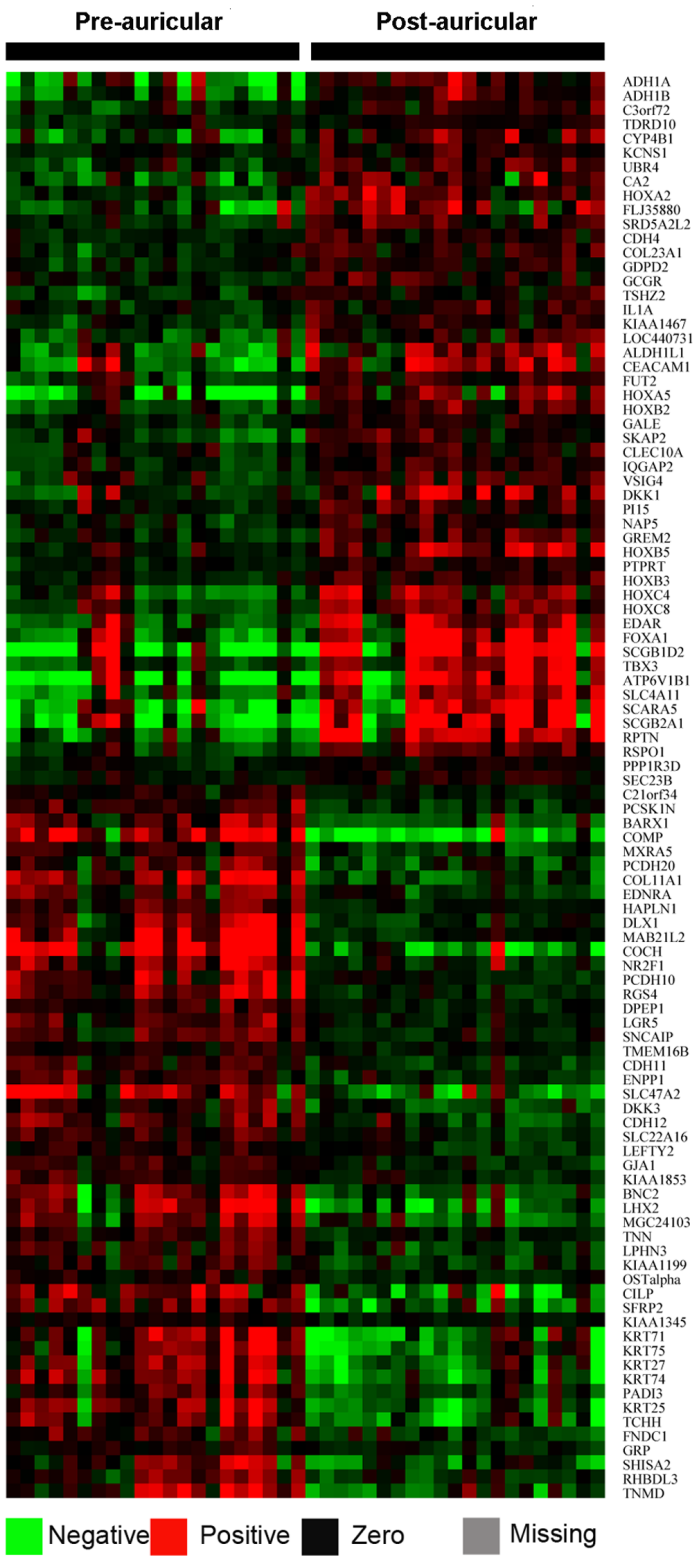
Heatmap of the top 100 shared DEGs identified by both GeneSpring and GSEA. Group names are on top and gene symbols on the right.

The DEGs between sun-exposed pre-auricular and sun-protected post-auricular skin samples were identified mainly with GeneSpring GX 10.0. Of the 37,352 examined probes, 13,950 (37%) were expressed under our criteria. Among these expressed probes, 1,762 probes (corresponding to 1,621 genes) were differentially expressed at a cutoff of *Asymptotic* P-value < 0.05 by an *unpaired T-test* (Table S1). Of the 1,621 genes, 756 (47 %) were up-regulated, and 865 (53 %) were down-regulated in photoaging. To compare the differences in expression between pre-auricular and post-auricular skin tissues, we display the top 100 shared DEGs identified by both GeneSpring and GSEA in Figure 1. Good agreement was observed between the two different analyses (Table S2). This indicates the reliability of the findings between pre-auricular and post-auricular skin tissues.

To uncover photoaging-related biological processes, we conducted the functional enrichment analyses using three different software packages, WebGestalt, DAVID, and GSEA. These three software packages are integrated with functional annotation databases and contain various functional analysis modules for different biological contexts. Two functional annotation databases, GO and KEGG, were used for most of our functional analyses. We considered the shared terms from the three software packages as meaningful enrichment in photoaging.

### GO Enrichment Analysis

To identify the significantly enriched GO terms, we used the modules of “GO Tree”, “GO Functional Annotation Chart”, and “all of the GO gene sets”, implemented in WebGestalt, DAVID, and GSEA, respectively. Table 3 displays the shared enriched GO terms with significance levels of p<0.01 in WebGestalt, p<0.05 in DAVID, and FDR<0.25 in GSEA. Both the up-regulated and down-regulated GO terms in pre-auricular skin were further divided into three sub-categories: biological progress, cell component, and molecular function. In the biological progress sub-category, the up-regulated GO terms were mainly related to signaling and development (e.g., the GO terms of *transforming growth factor beta receptor signaling pathway* and *epidermis development*); whereas most of the down-regulated GO terms were related to metabolism (e.g., the GO term of *lipid metabolic process*). In the cell component sub-category, the up-regulated GO terms were mainly related to extracellular matrix and intermediate filament cytoskeleton (e.g., the GO terms of *extracellular matrix* and *intermediate filament cytoskeleton*); whereas the down-regulated GO terms were mainly related to endoplasmic reticulum and membrane (e.g., the GO terms of *endoplasmic reticulum* and *membrane faction*). Lastly, in the molecular function sub-category, there were no common up-regulated GO terms; and most of the down-regulated GO-terms were related to the catalytic activity molecular function (e.g., the GO term of *oxidoreductase activity*).

**Table 3:** Common enriched GO terms by the three software packages: WebGestalt, DAVID and GSEA.

Regulation*	Sub-category#	Go term	P-value by WebGestalt	P-value by DAVID	FDR by GSEA
**Up**	**BP**	transmembrane receptor protein serine threonine kinase signaling pathway	4.28e-4	1.43e-3	0.15
		transforming growth factor beta receptor signaling pathway	6.83e-4	3.23e-4	0.17
		DNA replication	7.20e-4	4.54e-3	0.15
		epidermis development	5.32e-4	1.15e-6	0.18
		tissue development	3.48e-3	2.23e-7	0.20
		ectoderm development	1.09e-3	1.20e-6	0.20
		organ development	2.03e-3	3.78e-8	0.23
	**CC**	extracellular matrix	1.48e-6	1.26e-6	0.08
		extracellular matrix part	1.27e-4	6.63e-5	0.10
		extracellular region part	5.76e-5	1.27e-4	0.22
		basement membrane	3.21e-3	3.64e-2	0.15
		cytoskeletal part	4.83e14	4.75e-15	0.22
		intermediate filament cytoskeleton	4.74e-26	9.72e-33	0.21
		intermediate filament	4.74e-26	3.07e-32	0.22
		replication fork	5.14e-4	3.77e-2	0.17
	**MF**	NA	NA	NA	NA
**Down**	**BP**	lipid metabolic process	7.75e-11	1.3e-14	0.11
		carboxylic acid metabolic process	1.15e-10	2.29e-12	0.05
		organic acid metabolic process	1.36e-10	3.22e-12	0.06
		cellular lipid metabolic process	4.10e-11	3.58e-11	0.07
		lipid biosynthetic process	1.06e-9	4.49e-10	0.08
		fatty acid metabolic process	1.46e-9	5.68e-8	0.07
		carbohydrate metabolic process	1.31e-4	5.57e-7	0.17
		steroid metabolic process	3.37e-4	7.84e-5	0.12
		generation of precursor metabolites and energy	1.85e-7	5.26e-4	0.13
		fatty acid oxidation	7.95e-5	5.99e-4	0.10
		glycolipid metabolic process	1.93e-3	7.15e-4	0.15
		steroid biosynthetic process	2.14e-4	1.06e-3	0.96
		excretion	1.43e-5	5.60e-4	0.06
		amine catabolic process	3.52e-5	5.50e-3	0.06
	**CC**	endoplasmic reticulum	3.35e-4	1.41e-7	0.17
		endoplasmic reticulum part	5.16e-3	4.65e-7	0.20
		cell fraction	2.06e-4	4.92e-7	0.19
		membrane fraction	3.38e-5	1.27e-6	0.20
		intrinsic to organelle membrane	1.38e-5	2.57e-6	0.15
		endoplasmic reticulum membrane	1.82e-3	9.08e-6	0.17
		intrinsic to endoplasmic reticulum membrane	7.22e-5	1.12e-5	0.11
		organelle membrane	1.45e-3	7.82e-5	0.20
		integral to organelle membrane	1.38e-5	1.06e-4	0.21
		integral to endoplasmic reticulum membrane	4.61e-3	1.87e-3	0.22
		nuclear envelope-endoplasmic reticulum network	2.92e-3	1.04e-5	0.19
	**MF**	oxidoreductase activity	2.12e-10	4.75e-12	0.08
		lyase activity	1.10e-3	1.70e-4	0.22
		monooxygenase activity	2.16e-3	9.68e-4	0.14
		anion channel activity	3.05e-3	3.88e-2	0.11

* Up, up-regulation; Down, down-regulation.

# BP, biological progress; CC, cell component; MF, molecular function.

NA, not available.

### KEGG Pathway Enrichment Analysis

To identify the significantly enriched KEGG pathways, we used the modules “KEGG Table and Maps”, “KEGG Functional Annotation Chart”, and “KEGG pathways gene sets”, implemented in WebGestalt, DAVID, and GSEA, respectively. The shared enriched pathways (Table 4) were determined at the significance levels p<0.01 in WebGestalt, p<0.1 in DAVID, and FDR<0.2 in GSEA, respectively. Of the 11 enriched pathways, three were up-regulated and nine were down-regulated in photoaging. The three up-regulated pathways were *ECM-receptor interaction*, *TGF-beta signaling pathway*, and *Cell cycle*. All of the down-regulated pathways were metabolic-related and covered various types of metabolic pathways, such as amino acid metabolism (n = 3), carbohydrate metabolism (n = 2), lipid metabolism (n = 1), glycan biosynthesis and metabolism (n = 1), and energy metabolism (n = 1). Similar biological pathways were also found with the use of other pathway annotation databases by DAVID or GSEA (Table 5). Furthermore, good concordance was observed between the enriched functional modules identified by the KEGG and GO enrichment analyses (e.g., th*e* KEGG pathway *TGF-beta signaling pathway* and the GO term *transforming growth factor beta receptor signaling pathway*).

**Table 4:** Common enriched KEGG pathways by the three software packages: WebGestalt, DAVID and GSEA.

Regulation	Pathway name	P-value by WebGestalt	P-value by DAVID	FDR by GSEA
**Up-regulation**	ECM-receptor interaction	7.86e-7	1.72e-4	0.16
	TGF-beta signaling pathway	3.37e-5	4.15e-3	0.13
	Cell cycle	1.87e-3	5.12e-2	0.13
**Down-regulation**	Valine, leucine and isoleucine degradation	1.77e-7	2.39e-4	0.13
	Arginine and proline metabolism	3.72e-6	1.44e-2	0.10
	Fructose and mannose metabolism	1.27e-5	3.46e-3	0.09
	Glycolysis / Gluconeogenesis	1.09e-6	5.80E-2	0.14
	Glycosphingolipid biosynthesis – ganglio series	1.20e-5	7.08e-3	0.12
	Tryptophan metabolism	1.84e-5	3.67e-2	0.15
	Arachidonic acid metabolism	4.17e-5	3.56e-2	0.11
	Nitrogen metabolism	2.09e-4	4.76e-2	0.14

**Table 5:** The related enriched pathways in other databases.

Regulation*	Pathway database	Pathway name	P-value/FDR#	Software package
**Up**	REACTOME_PATHWAY	Cell cycle, Mitotic	8.88e-4	DAVID
	REACTOME_PATHWAY	Signaling by BMP	3.32e-2	DAVID
	GenMAPP	DNA replication reactome	0.23	GSEA
	GenMAPP	G1 to S cell cycle reactome	0.25	GSEA
	BIOCARTA	Cell cycle:G2/M Checkpoint	0.03	GSEA
	BIOCARTA	Effects of calcineurin in Keratinocyte Differentiation	0.11	GSEA
	BIOCARTA	Cell cycle:G1/S Checkpoint	0.13	GSEA
	BIOCARTA	Cyclins and Cell Cycle Regulation	0.14	GSEA
**Down**	BIOCARTA	Catabolic Pathways for Arginine, Histidine, Glutamate, Glutamine, and Proline	3.69e-2	DAVID
	REACTOME_PATHWAY	Metabolism of amino acids	7.85e-4	DAVID
	REACTOME_PATHWAY	Metabolism of carbohydrates	8.25e-4	DAVID
	REACTOME_PATHWAY	Metabolism of lipids and lipoproteins	8.81e-4	DAVID
	GenMAPP	Arginine and proline metabolism	0.07	GSEA
	GenMAPP	Nitrogen metabolism	0.10	GSEA
	GenMAPP	Tryptophan metabolism	0.08	GSEA
	GenMAPP	Glycolysis and gluconeogenesis	0.10	GSEA
	GenMAPP	Glycolysis	0.14	GSEA
	GenMAPP	Gluconeogenesis	0.14	GSEA
	GenMAPP	Fructose and mannose metabolism	0.13	GSEA
	GenMAPP	Valine leucine and isoleucine degradation	0.15	GSEA

* Up, up-regulation; Down, down-regulation.

# P-value for results by DAVID, FDR for results by GSEA.

**Figure 2:**
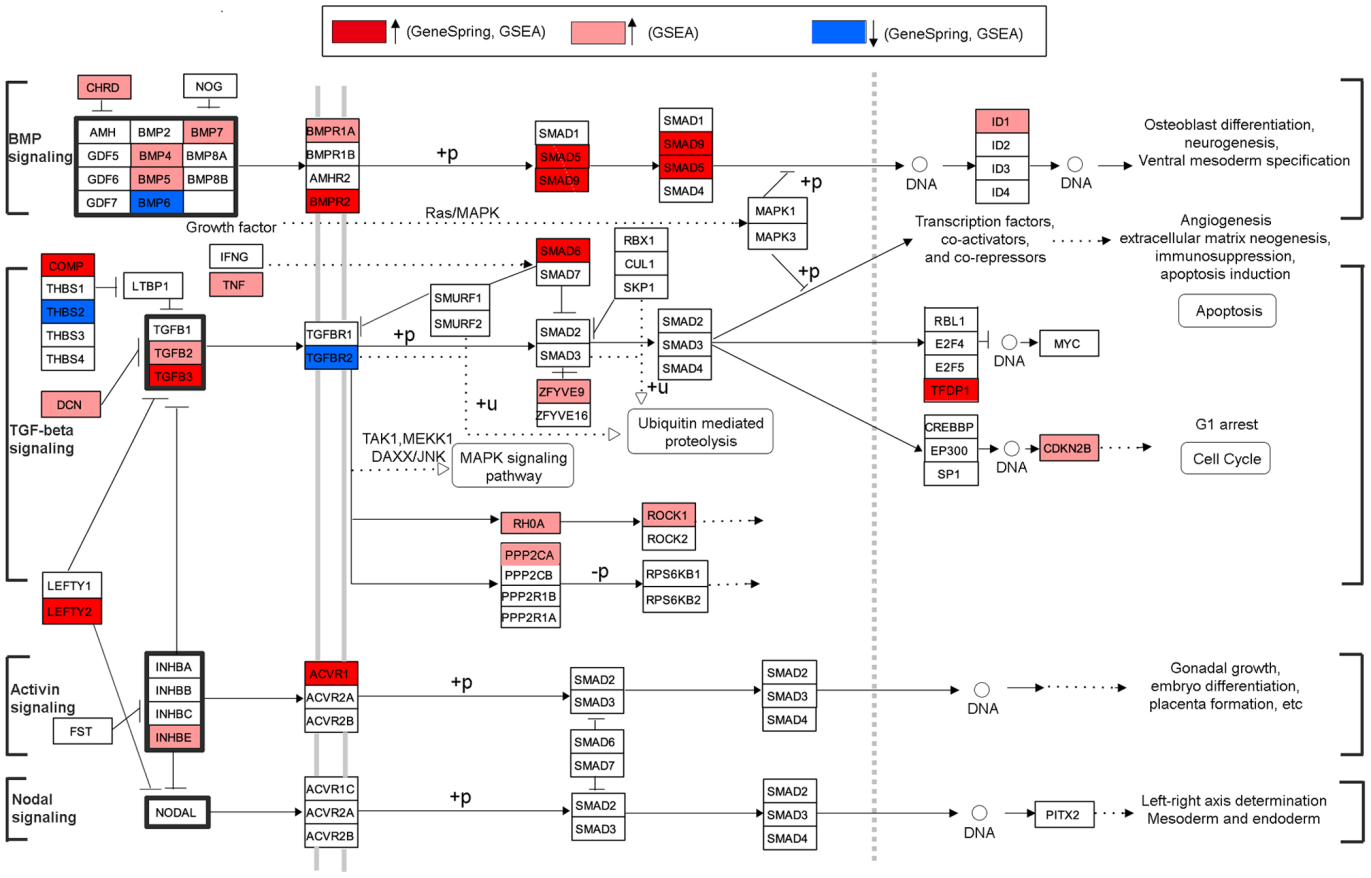
Modified “TGF-beta signaling pathway” from KEGG. Protein symbols were replaced by gene symbols to reflect gene-based data. For better legibility, gene symbols were not italicized. See the online information http://www.genome.jp/kegg/document/help_pathway.html for detailed KEGG pathway map notation.

Comparing to GO, the KEGG pathway analysis provides biological information in a more detailed and specific manner. Therefore, we looked into two enriched KEGG pathways, one up-regulated and the other down-regulated, in detail. The *TGF-beta signaling pathway* is one of the three up-regulated pathways mentioned above. It is not only the most significant pathway identified in our study, but was also reported to be biologically relevant to photoaging [10], [18]–[20]. This signaling pathway interacts with the other two up-regulated pathways. Since the KEGG pathways are annotated as protein-based pathways, multiple genes may correspond to one protein in our gene-based microarray analysis. To have a clear and informative view, we annotated the pathway map in genes instead of proteins. In Figure 2, all the DEGs in the *TGF-beta signaling pathway* are color-coded (red for up-regulation and blue for down-regulation). As we observed, most of the DEGs in this pathway were up-regulated rather than down-regulated.

**Figure 3:**
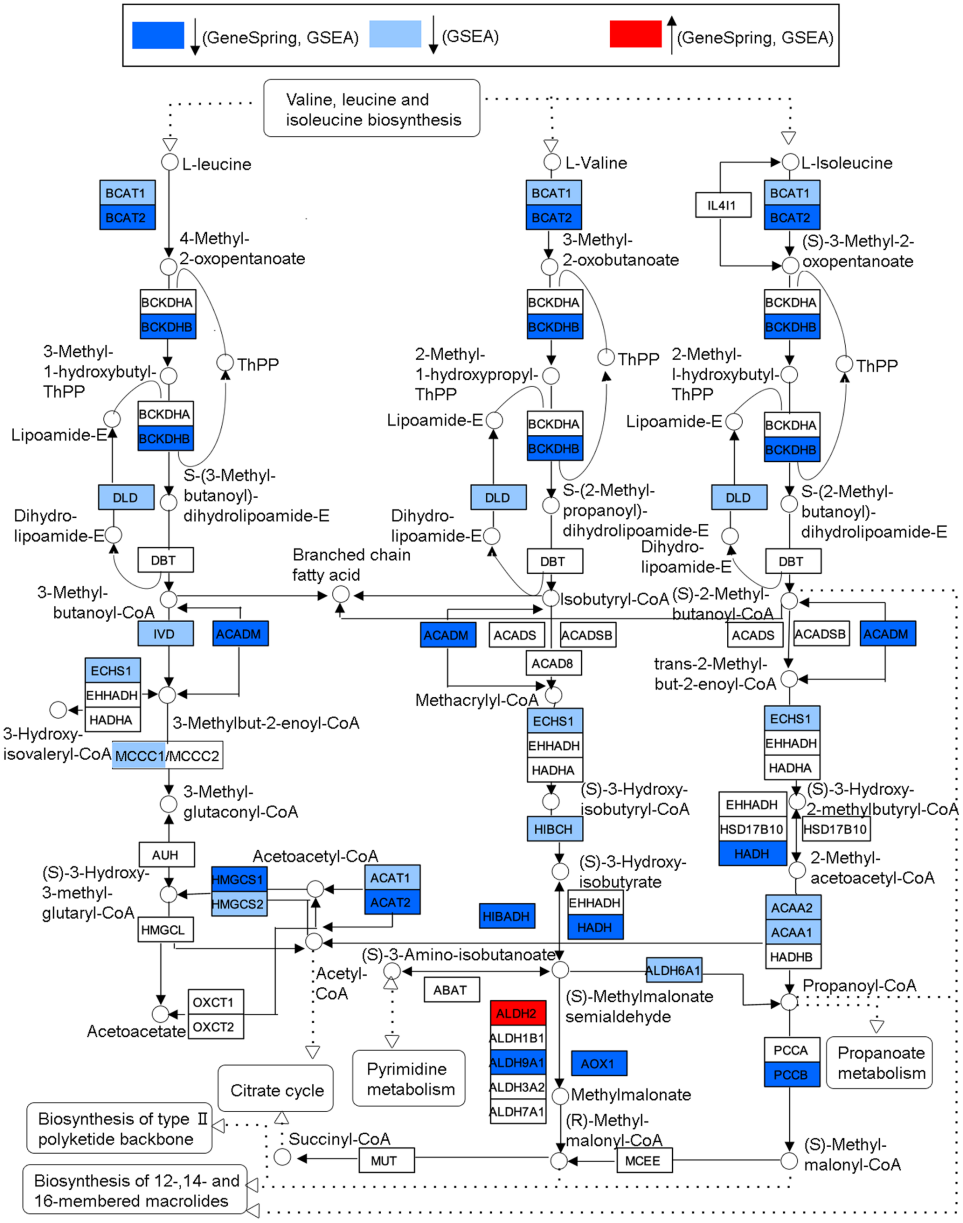
Modified “Valine, leucine and isoleucine degradation” pathway from KEGG. Protein symbols were replaced by gene symbols to reflect gene-based data. For better legibility, gene symbols were not italicized. See the online information http://www.genome.jp/kegg/document/help_pathway.html for detailed KEGG pathway map notation.

The down-regulated pathways were all metabolic-related and covered various types of metabolisms. This indicated that photoaging has a negative effect on the skin metabolism. To illustrate this metabolic change, we used the enriched *Valine, leucine and isoleucine degradation* pathway from the KEGG analysis as an example (see Figure 3). We derived a pathway map of *Valine, leucine and isoleucine degradation* in a similar way to that of the *TGF-beta signaling pathway* map. It is obvious that almost all of the DEGs were down-regulated in this pathway with only one exception (*ALDH2*).

### Quantitative Real-time PCR

**Figure 4:**
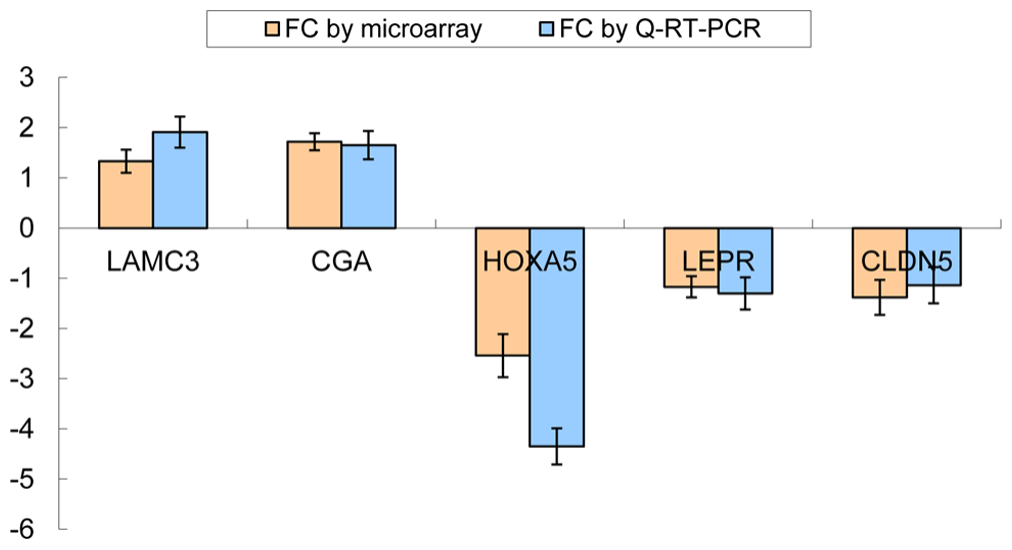
Expression concordance between microarray and Q-RT-PCR assays. The X-axis indicates the examined genes. The Y-axis represents the fold changes (FC) of expression values between the pre-auricular skin and post-auricular skin. For better legibility, gene symbols were not italicized.

We selected five genes (*HOXA5, LEPR, CLDN5, LAMC3*, and *CGA*) to validate the expression data from microarray analysis using SYBR-green based Q-RT-PCR. The expression of these genes was significantly altered in various functional modules. For example, *LAMC3* is involved in the pathway of *ECM-receptor interaction, LEPR* is involved in *steroid metabolic processes* and *CGA* is a member of the a*mino acids metabolism* process. The expression ratios of the five genes, as determined by both microarray and Q-RT-PCR, are shown in Figure 4. Good agreement was observed between the two platforms.

## Discussion

With global gene expression profiling, this study uncovered DEGs from paired sun-exposed and sun-protected skin tissues of Chinese women. Subsequently, functional enrichment analyses were performed on the DEGs to identify photoaging-related biological processes.

### Up-regulated Functional Modules in Sun-exposed Pre-auricular Skin

From enrichment analyses of up-regulation in sun-exposed skin, two shared functional modules, TGF-β signaling pathway and cell cycle/related process (DNA replication), were observed between the GO and KEGG analyses, whereas the unshared modules were functionally related to these two modules. The TGF-β family signaling pathway (as the KEGG-assigned *TGF-beta signaling pathway*) comprises a group of pathways mediated by the TGF-β superfamily ligands, such as bone morphogenetic Proteins (BMPs), TGF-βs, Activins, Nodal, and growth and differentiation factors (GDFs) (Figure 2). Each class of ligands mediates a specific type of signaling. Based on the type of intracellular mediators (regulatory-Smads; R-Smads), the TGF-β family signaling pathway can be divided into two major groups: signaling through Smad-2 /3 with ligands as TGF-βs, activins, and nodal, and signaling through Smad-1/5/8 with ligands as BMPs and GDFs [21].

The observed up-regulation of the TGF-β family signaling pathway was an overall assessment of the expression of all genes in this signaling family. This overall observation may not reflect the exact expression of each individual pathway regarding photoaging. In particular, TGF-β signaling was reported to be responsible for the process of photoaging in an inactivated manner [10], which is contrary to our overall finding of up-regulation. In TGF-β signaling, of 13 genes found differentially regulated in our study (Figure 2); 11 were up-regulated and 2 were down-regulated. Interestingly, 6 genes (*DCN, LEFTY2, INHBE, SMAD6, TNF,* and *TFDP1*) out of the 11 up-regulated genes were negative regulators. Together with the 2 down-regulated genes (*TGFBR2* and *THBS1*), which contribute positively to TGF-β signaling, this complex finding seems to agree with reported down-regulation of TGF-β signaling. Furthermore, various studies demonstrated that UV could inhibit TGF-β signaling through the *TGFBR2* gene [18]–[20]. TGFBR2 directly binds to TGF-βs and mediates a critical step of TGF-β signaling. The receptor was found to be significantly decreased at both the mRNA [10], [18]–[20] and protein levels [10], [19], [20] in acute UV damage and photoaging. Quan *et al*. demonstrated that the down-regulation of TGFBR2 resulted in 90% reduction in the binding of TGF-β [20] and suggested a major role in the repression of TGF-β signaling [19]. Aside from the above canonical TGF-β signaling (TGF-β/Smad signaling), we also observed up-regulation of the TGF-β/RhoA and TGF-β/PP2A signaling pathways. However, their biological functions in photoaged skin are not clear to us. It was known that TGF-β signaling could regulate multiple cellular functions, such as apoptosis, cell cycle, extracellular matrix assembly [22], [23]. The specific roles of TGF-β signaling in photoaging remain elusive.

BMP signaling is the best known example of Smad-1/-5/-8-mediated signaling from the TGF-β family signaling pathway. We found that numerous genes in the pathway were up-regulated, except for *BMP6* (Figure 2). A recent study demonstrated the involvement of BMP signaling in regulating epidermal homeostasis (proliferation and differentiation), melanogenesis, and hair follicle growth in postnatal skin [24]. Notably, our results on up-regulation, such as GO term *epidermis development* (Table 3) and the BIOCARTA pathway *Effects of calcineurin in Keratinocyte Differentiation* (Table 5), are related to the epidermis homeostasis. Additionally, studies reported that UV could affect epidermis progresses, such as keratinocyte proliferation and differentiation [9], [25]. Although not enough evidence of the involvement of BMP signaling in photoaging was found, our results support the notion that BMP signaling may function in photoaging at least partially by regulating the epidermal homeostasis.

In this study, the other functional modules enriched in up-regulation are functionally related to the TGF-β family signaling pathway. The processes of cell cycle and ECM neogenesis interact with the TGF-β family signaling pathway as seen in Figure 2. Our enriched KEGG pathway *ECM-receptor interaction* can function as the TGF-β family signaling pathway to regulate cell cycle and apoptosis through the p70S6K-cyclin-D1 pathway and/or the mediation of p53 and Rb [26]. Also, the enriched *epidermis development*-related GO terms are functionally related to the actions of BMP signaling. Collectively, the up-regulated functional modules play important roles in cell proliferation, differentiation, apoptosis, etc. Alterations of these processes can affect skin homeostasis.

### Down-regulated Functional Modules in Sun-exposed Pre-auricular Skin

According to both the GO and KEGG analyses in our photoaging study, the down-regulated functional modules were all related to metabolic processes. The altered skin metabolisms extended a wide range of categories, including amino acid metabolism, carbohydrate metabolism, lipid metabolism, energy metabolism, and glycan biosynthesis and metabolism.

Most of our enriched metabolic processes were related to energy production, such as *Glycolysis/Gluconeogenesis* (a KEGG pathway) and *generation of precursor metabolites and energy* (a GO term). Notably, some amino acid catabolism pathways, such as *Valine, leucine and isoleucine degradation, Arginine and proline metabolism* (the top two down-regulated KEGG pathways*)*, and *Tryptophan metabolism* were identified in down-regulation. A possible explanation is that the amino acid catabolism may also provide precursors to energy production, and down-regulation of these pathways may further affect the energy production of skin cells. Figure 3, the *Valine, leucine and isoleucine degradation* pathway, is used to illustrate the down-regulation. A striking down-regulation for all DEGs in this pathway, except for *ALDH2,* is noticed. Studies have reported the mtDNA deletions in photoaged skin [27], [28]. Berneburg *et al.* indicated that these mtDNA deletions may lead to the decline of the energy production of mitochondria [29]. Jacobson *et al.* found that UV radiation can disrupt cellular energy metabolism at multiple levels [30]. Wilson and Morley also indicated that the decreased energy metabolism can be induced by chronological aging [31]. These reports and our findings support that energy production is decreased in photoaged skin.

In addition to serving energy production, the amino acid catabolism (in particular, the trytophan metabolism) can provide precursors for the synthesis of some important nitrogenous compounds, such as serotonin. Our results detected down-regulation of the trytophan metabolism with both KEGG and GenMAPP analyses. The trytophan metabolism can lead to production of serotonin and subsequently melatonin, both reported to affect cell proliferation [32], [33]. Melatonin has properties of anti-oxidant and free radicals scavenger [32], [34]. Moreover, serotonin and melatonin were known to be in the cutaneous neuroendocrine system, which involve in the regulation of skin and body homeostasis [34]. Therefore, it is possible that photoaging may indirectly disrupt the integrity and homeostasis of the skin through affecting the production of serotonin and melatonin by the down-regulation of the trytophan metabolism.

The lipids of skin tissues are essential for several skin functions, such as epidermal barrier homeostasis, energy metabolism, and cell growth and differentiation [35], [36]. Down-regulation of the lipid metabolism was observed here and reported in other photoaging studies. Robinson *et al.* conducted one of the two previously mentioned genome-wide studies in photoaging. They observed down-regulation of the lipid-metabolism-related GO terms (*lipid biosynthetic process* and *cholesterol metabolic process*) [12]. Kim *et al.* also reported photoaging-related decreases in the levels of free fatty acids and triglycerides and in gene expression regarding lipid synthesis [11].

In the lipid metabolism, down-regulation of steroid metabolic/biosynthetic processes may affect the function of cholesterol, steroids, and vitamin D. Previous studies demonstrated that the skin can produce steroid, vitamin D3 and vitamin D3-hydroxyderivatives through cholesterol [34], [37], [38]. Vitamin D3 and vitamin D3-hydroxyderivatives can modulate cell proliferation and differentiation [37], [38] and further influence the tissue or organ homeostasis. Also, other findings reported that the skin as a neuroendocrine organ can contribute to the maintenance of skin and body homeostasis through steroids [34], [39], [40]. Collectively, the observation of down-regulated steroid metabolic/biosynthetic processes suggests that the photoaging process may disrupt the skin and body homeostasis.

Other than this study, there are two photoaging-related studies at the global level of gene expression. The first study, performed by Urschitz *et al.* [9], found 25 differentially expressed UniGenes, but 5 of them were deleted later from the UniGene database. Of the remaining 20 UniGenes, 3 (*C3orf10*, *COMP*, and *RPL37*) were shared with our findings. The second study, performed by Robinson *et al.* [12] with skin tissues of extensor forearms from older vs. younger women, reported similar results to ours in the down-regulated GO terms, such as *lipid biosynthetic process* and *steroid biosynthetic process*. However, the up-regulated GO terms, such as *response to wounding* and *immune response,* were not found in our study. The discrepancy in findings between these studies and ours may be partly due to the difference in ethnicity of sample population. It is known that ethnic groups with different skin tones respond to solar radiation differently [13]. Asian populations, have a darker skin tone than that of the Europeans used by the two other studies. Additionally, differences in sample size, sample collection, method for expression assay, and statistical analysis tools may also contribute to the different findings.

To conclude our findings in photoaging, the up-regulated functional modules are mostly related to the regulation of cell fate, such as cell proliferation, differentiation, and apoptosis. This suggests that skin homeostasis is disturbed due to photoaging. The down-regulated functional modules are mostly metabolic-related, and may affect the energy production as well as the homeostasis of skin and body. These findings provide a foundation for future prevention, treatment, and molecular research in photoaging.

## Supporting Information

Figure S1 **Performance of quantile normalization.** The X-axis indicates samples used in the present study, and the IDs are the raw Illumina BeadChip IDs.
(TIF)

Table S1 **List of the 1,762 differentially expressed probes.**
(PDF)

Table S2 **List of the top 100 shared DEGs identified by both GeneSpring and GSEA.**
(PDF)
