# Supplementary figures and images for: Transcriptome Analysis of Skin Photoaging in Chinese Females Reveals the Involvement of Skin Homeostasis and Metabolic Changes
Source: PLoS One. 2013 Apr 24;8(4):e61946. doi: 10.1371/journal.pone.0061946 (PMC3634825; doi:10.1371/journal.pone.0061946)

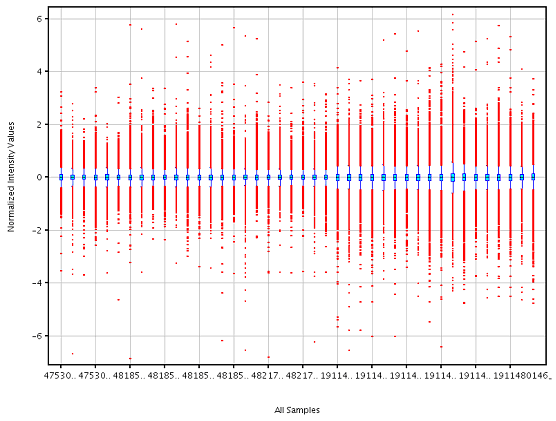

Supplement: Figure S1 — Performance of quantile normalization. The X-axis indicates samples used in the present study, and the IDs are the raw Illumina BeadChip IDs. (TIF) [file pone.0061946.s001.tif]
