# Supplementary material for: Transcriptome Analysis of Skin Photoaging in Chinese Females Reveals the Involvement of Skin Homeostasis and Metabolic Changes
Source: PLoS One. 2013 Apr 24;8(4):e61946. doi: 10.1371/journal.pone.0061946 (PMC3634825; doi:10.1371/journal.pone.0061946)
Supplement: Table S1 — List of the 1,762 differentially expressed probes. (PDF) [file pone.0061946.s002.pdf]

Probe_Id	Symbol	P-value	Regulation	Accession
ILMN_1769118	9-Sep	6.76E-04	down	NM_006640.3
ILMN_1735045	A4GALT	0.04910979	down	NM_017436.4
ILMN_1676336	AADACL1	0.043147337	down	NM_020792.3
ILMN_1701551	ABCA6	0.028997697	down	NM_080284.2
ILMN_1812070	ABCB1	0.022279972	down	NM_000927.3
ILMN_1776119	ABCC10	0.005977289	down	NM_033450.2
ILMN_1788457	ABCC4	0.003766991	down	NM_005845.2
ILMN_1810085	ABCF3	3.43E-04	down	NM_018358.2
ILMN_1745116	ABHD12	0.027677927	down	NM_015600.3
ILMN_1706344	ABHD6	0.040046554	down	NM_020676.4
ILMN_1731433	ABP1	0.005207185	down	NM_001091.2
ILMN_1753370	ABTB2	0.003175984	down	NM_145804.1
ILMN_1763852	ACACB	0.02344228	down	NM_001093.3
ILMN_1775579	ACAD9	0.005419644	down	NM_014049.3
ILMN_1660890	ACADL	0.00918695	down	NM_001608.2
ILMN_1778104	ACADM	0.016041834	down	NM_000016.2
ILMN_1806408	ACADVL	0.03166072	down	NM_001033859.1
ILMN_1708672	ACAT2	0.029056797	down	NM_005891.2
ILMN_1750800	ACO1	0.043151073	down	NM_002197.1
ILMN_1685703	ACOX2	0.039346	down	NM_003500.2
ILMN_1714197	ACSS2	0.003291502	down	NM_018677.2
ILMN_1671703	ACTA2	0.007660472	down	NM_001613.1
ILMN_1803686	ADA	0.023124892	down	NM_000022.2
ILMN_1793040	ADAMTSL5	0.03270107	down	NM_213604.1
ILMN_1698777	ADCK1	0.002102097	down	NM_020421.2
ILMN_1655935	ADCY7	0.013245398	down	NM_001114.3
ILMN_1764309	ADH1A	1.63E-04	down	NM_000667.2
ILMN_1811598	ADH1B	2.80E-05	down	NM_000668.3
ILMN_1702858	ADHFE1	0.004988652	down	NM_144650.2
ILMN_1712944	AES	0.047236606	down	NM_001130.5
ILMN_1814327	AGTR1	0.005892068	down	NM_000685.4
ILMN_1770412	AHCYL1	0.007035109	down	NM_006621.4
ILMN_1668408	AIFM1	0.003401587	down	NM_145813.1
ILMN_1669557	AIM1L	0.0261749	down	NM_001039775.1
ILMN_1677043	AKR7A2	0.027147416	down	NM_003689.2
ILMN_1700047	ALAS1	0.047573555	down	NM_000688.4
ILMN_1802167	ALDH1L1	3.88E-05	down	NM_012190.2
ILMN_1656368	ALDH4A1	0.00241245	down	NM_170726.1
ILMN_1761804	ALDH9A1	0.021833396	down	NM_000696.3
ILMN_1741148	ALDOA	0.021229371	down	NM_184043.1
ILMN_1713731	ALOX12	0.002009921	down	NM_000697.2
ILMN_1783443	ALOX15	0.007021188	down	XM_001131480.1
ILMN_1751975	ALOX15B	0.028343659	down	NM_001141.2
ILMN_1678255	ALOXE3	0.001763468	down	NM_021628.1
ILMN_1702322	ALS2CL	0.024319489	down	NM_147129.2
ILMN_1774447	AMPD3	0.00498151	down	NM_000480.2

ILMN_1797873	ANKK1	0.017104354	down	NM_178510.1
ILMN_1756417	ANKRD37	0.009047979	down	NM_181726.2
ILMN_1763837	ANPEP	0.0057603	down	NM_001150.1
ILMN_1767113	AOX1	0.001758385	down	NM_001159.3
ILMN_1796737	APM-1	0.03677468	down	XM_113971.4
ILMN_1789007	APOC1	0.011010014	down	NM_001645.3
ILMN_1807036	AQP5	0.008546386	down	NM_001651.1
ILMN_1731287	ARFGAP3	0.005391518	down	NM_014570.3
ILMN_1800898	ARG2	0.003915094	down	NM_001172.3
ILMN_1782761	ARHGAP20	0.031550698	down	NM_020809.2
ILMN_1697118	ARMC6	0.04765612	down	NM_033415.2
ILMN_1664001	ARSF	0.002523565	down	NM_004042.3
ILMN_1748970	ATAD4	0.005546042	down	NM_024320.2
ILMN_1804445	ATF7IP2	9.87E-04	down	NM_024997.2
ILMN_1734655	ATG9B	0.042145915	down	NM_173681.4
ILMN_1654065	ATOH8	3.49E-04	down	NM_032827.4
ILMN_1658071	ATP1B1	0.001007554	down	NM_001677.3
ILMN_1697827	ATP2A3	0.02020054	down	NM_174955.1
ILMN_1772132	ATP5B	0.030364258	down	NM_001686.3
ILMN_1678186	ATP6V0A4	0.00298181	down	NM_020632.2
ILMN_1789005	ATP6V0C	0.041513342	down	XM_001130742.1
ILMN_1812073	ATP6V1B1	1.49E-04	down	NM_001692.3
ILMN_1783956	ATP8B4	0.01043012	down	NM_024837.2
ILMN_1727390	ATPAF2	0.001069399	down	NM_145691.3
ILMN_1705900	ATXN10	0.001444084	down	NM_013236.2
ILMN_1797154	AZGP1	0.001139725	down	NM_001185.2
ILMN_1766221	B4GALT1	0.04804083	down	NM_001497.2
ILMN_1703593	BAIAP2L1	0.033925988	down	NM_018842.3
ILMN_1695110	BCAT2	0.00573152	down	NM_001190.2
ILMN_1764166	BCKDHB	0.003463642	down	NM_183050.1
ILMN_1670398	BCR	0.04161546	down	NM_004327.3
ILMN_1738529	BCS1L	0.001553867	down	NM_001079866.1
ILMN_1799280	BDH1	0.00905534	down	NM_203314.2
ILMN_1684086	BDKRB2	0.002249889	down	NM_000623.2
ILMN_1755796	BEST2	0.019529361	down	NM_017682.2
ILMN_1813043	BHMT2	0.015197326	down	NM_017614.3
ILMN_1770505	BIK	0.009087113	down	NM_001197.3
ILMN_1747650	BMP6	0.048419558	down	NM_001718.4
ILMN_1699728	BTD	0.02015177	down	NM_000060.2
ILMN_1680110	C10orf116	0.021433331	down	NM_006829.2
ILMN_1674373	C10orf27	8.40E-04	down	NM_152710.2
ILMN_1765232	C10orf59	0.034337383	down	NM_018363.2
ILMN_1797104	C10orf95	0.042304184	down	NM_024886.1
ILMN_1684357	C14orf139	0.045124806	down	XR_017875.1
ILMN_1773017	C14orf73	0.008611272	down	NM_001077594.1
ILMN_1815812	C16orf5	0.011947041	down	NM_013399.2
ILMN_1729450	C16orf62	0.025444044	down	NM_020314.4

ILMN_1759411	C16orf79	0.00980263	down	NM_182563.2
ILMN_1697800	C16orf86	0.02923869	down	NM_001012984.2
ILMN_1702676	C17orf68	2.33E-04	down	XM_938283.2
ILMN_1755711	C17orf68	7.33E-04	down	XM_938283.2
ILMN_1678052	C19orf24	0.049850848	down	NM_017914.2
ILMN_1813374	C19orf28	0.003009261	down	NM_174983.3
ILMN_1801697	C19orf46	0.001293929	down	NM_001039876.1
ILMN_1674817	C1orf115	0.009551729	down	NM_024709.4
ILMN_1706483	C1orf116	2.78E-04	down	NM_023938.5
ILMN_1786273	C1orf122	0.004175028	down	NM_198446.1
ILMN_1733298	C1orf158	0.046677444	down	NM_152290.1
ILMN_1661595	C1orf53	0.016801553	down	NM_001024594.1
ILMN_1796409	C1QB	0.001377066	down	NM_000491.3
ILMN_1785902	C1QC	0.003253549	down	NM_172369.2
ILMN_1694608	C1QTNF1	0.035793368	down	NM_198593.1
ILMN_1797828	C20orf116	0.007416161	down	NM_023935.1
ILMN_1689585	C20orf194	0.008878233	down	NM_001009984.1
ILMN_1740923	C20orf54	6.65E-04	down	NM_033409.2
ILMN_1652512	C2CD2	0.01640185	down	NM_015500.1
ILMN_1676822	C2orf40	0.002423857	down	NM_032411.1
ILMN_1787529	C3AR1	0.03219087	down	NM_004054.2
ILMN_1792415	C3orf39	0.013698746	down	NM_032806.4
ILMN_1694470	C3orf72	1.82E-05	down	NM_001040061.1
ILMN_1704637	C4orf23	0.001576767	down	NM_152544.1
ILMN_1761566	C5orf32	0.015036966	down	NM_032412.3
ILMN_1688242	C6	0.003491514	down	NM_000065.1
ILMN_1790461	C6orf125	0.047438834	down	NM_032340.2
ILMN_1752476	C6orf134	0.02787828	down	NM_024909.1
ILMN_1711203	C6orf15	0.004255861	down	NM_014070.2
ILMN_1655572	C6orf59	0.001048763	down	XR_017956.1
ILMN_1712616	C6orf81	0.04159322	down	NM_145028.3
ILMN_1790315	C7orf63	0.043515965	down	NM_001039706.1
ILMN_1781745	C9orf152	0.00177301	down	NM_001012993.1
ILMN_1763695	C9orf24	0.01913344	down	NM_147169.1
ILMN_1813837	C9orf9	0.023530878	down	NM_018956.3
ILMN_1662795	CA2	1.05E-04	down	NM_000067.1
ILMN_1708790	CA6	0.014361572	down	NM_001215.2
ILMN_1696615	CA8	0.0428553	down	NM_004056.4
ILMN_1783546	CARKD	0.023845442	down	NM_018210.2
ILMN_1718070	CASP9	0.002090548	down	NM_032996.1
ILMN_1811729	CBLC	0.02489748	down	NM_012116.2
ILMN_1813528	CCBE1	0.002290762	down	NM_133459.1
ILMN_1701477	CCDC101	0.028112557	down	NM_138414.1
ILMN_1709630	CCDC107	0.047965858	down	NM_174923.1
ILMN_1672759	CCDC109A	0.03662283	down	NM_138357.1
ILMN_1790555	CCDC146	0.010521263	down	NM_020879.2
ILMN_1799579	CCDC51	0.002450636	down	NM_024661.3

ILMN_1811050	CCDC88A	0.015489521	down	NM_018084.3
ILMN_1740609	CCL15	3.60E-04	down	NM_032964.2
ILMN_1677505	CCL21	0.002500319	down	NM_002989.2
ILMN_1701347	CCL28	0.011487394	down	NM_019846.3
ILMN_1774087	CCL28	0.006196046	down	NM_019846.3
ILMN_1667081	CCND2	0.029164175	down	NM_001759.2
ILMN_1776073	CCT4	0.027247513	down	NM_006430.2
ILMN_1740015	CD14	0.014088198	down	NM_000591.2
ILMN_1676372	CD209	4.94E-04	down	NM_021155.2
ILMN_1747622	CD33	0.017009486	down	NM_001772.3
ILMN_1784863	CD36	0.014286194	down	NM_001001548.1
ILMN_1727284	CD4	0.003415059	down	NM_000616.3
ILMN_1736567	CD74	0.03312101	down	NM_001025159.1
ILMN_1689989	CDC42BPB	0.017637847	down	NM_006035.3
ILMN_1724941	CDCP1	0.010330581	down	NM_022842.3
ILMN_1769891	CDH4	9.20E-07	down	NM_001794.2
ILMN_1716815	CEACAM1	1.51E-04	down	NM_001024912.1
ILMN_1670959	CEACAM5	0.016945807	down	NM_004363.2
ILMN_1712522	CEACAM6	2.90E-04	down	NM_002483.3
ILMN_1782050	CEBPD	0.037287388	down	NM_005195.3
ILMN_1654690	CECR5	0.035306305	down	NM_033070.2
ILMN_1800611	CEP72	0.029370341	down	NM_018140.3
ILMN_1777190	CFD	0.042998787	down	NM_001928.2
ILMN_1705813	CFTR	0.001628815	down	NM_000492.3
ILMN_1798083	CHERP	0.025004705	down	NM_006387.5
ILMN_1658504	CHKA	0.008163683	down	NM_212469.1
ILMN_1731353	CHPF	0.008563497	down	NM_024536.4
ILMN_1765257	CINP	0.032058857	down	NM_032630.2
ILMN_1764266	CKMT2	2.25E-04	down	NM_001825.2
ILMN_1787576	CLCNKA	0.002994274	down	NM_004070.3
ILMN_1685194	CLDN10	4.18E-04	down	NM_006984.3
ILMN_1721818	CLDN10	2.99E-04	down	NM_006984.3
ILMN_1708267	CLDN15	0.015743602	down	NM_138429.1
ILMN_1723042	CLDN3	0.042358723	down	NM_001306.2
ILMN_1728197	CLDN5	8.77E-04	down	NM_003277.2
ILMN_1653166	CLEC10A	7.17E-05	down	NM_006344.2
ILMN_1682176	CLEC3B	0.007319736	down	NM_003278.1
ILMN_1740502	CLEC4GP1	8.69E-04	down	NR_002931.1
ILMN_1669281	CLN3	0.036403086	down	NM_001042432.1
ILMN_1663538	CLYBL	0.005471535	down	NM_206808.1
ILMN_1712088	CLYBL	0.011910561	down	NM_206808.1
ILMN_1655720	CNNM4	0.04680986	down	NM_020184.3
ILMN_1761260	COBLL1	0.01677458	down	NM_014900.3
ILMN_1666665	COL23A1	1.04E-05	down	NM_173465.2
ILMN_1732151	COL6A1	0.03602805	down	NM_001848.2
ILMN_1736752	COMTD1	0.019090783	down	NM_144589.2
ILMN_1756898	COQ9	0.03595619	down	NM_020312.1

ILMN_1815634	COX4I2	0.04053037	down	NM_032609.2
ILMN_1755954	CPEB3	0.047024164	down	NM_014912.3
ILMN_1753910	CRISPLD1	0.03644758	down	NM_031461.3
ILMN_1790689	CRISPLD2	0.018639417	down	NM_031476.2
ILMN_1739956	CRSP2	0.00684568	down	XM_942919.1
ILMN_1805930	CSF1	0.02976729	down	NM_172212.1
ILMN_1686623	CSF1R	0.001428622	down	NM_005211.2
ILMN_1800354	CST3	0.008370932	down	NM_000099.2
ILMN_1679826	CST7	0.049580567	down	NM_003650.2
ILMN_1690880	CTAGE5	0.03714268	down	NM_203356.1
ILMN_1725090	CTHRC1	0.0326999	down	NM_138455.2
ILMN_1798360	CXCR7	0.016355306	down	NM_020311.2
ILMN_1724341	CXorf45	0.041892726	down	NM_001039210.1
ILMN_1812297	CYP26B1	0.019969035	down	NM_019885.2
ILMN_1758731	CYP2J2	0.004287477	down	NM_000775.2
ILMN_1810942	CYP3A5	0.046397515	down	NM_000777.2
ILMN_1659215	CYP4B1	1.31E-05	down	NM_000779.3
ILMN_1718603	CYP4F8	7.57E-04	down	NM_007253.3
ILMN_1702829	CYP4Z2P	0.012748781	down	XR_017801.1
ILMN_1722554	DCD	9.11E-04	down	NM_053283.2
ILMN_1740737	DCPS	0.025495877	down	NM_014026.3
ILMN_1681437	DCXR	0.005822702	down	NM_016286.2
ILMN_1737344	DDX41	0.048382703	down	NM_016222.2
ILMN_1686573	DEFB1	0.005173631	down	NM_005218.3
ILMN_1666503	DENND2A	0.017184794	down	NM_015689.2
ILMN_1692742	DENND3	0.013976004	down	NM_014957.2
ILMN_1761969	DERL2	0.016159596	down	NM_016041.3
ILMN_1681520	DGAT2	0.014033423	down	NM_032564.2
ILMN_1739830	DGAT2L6	0.010515923	down	NM_198512.1
ILMN_1789535	DHDDS	0.009021454	down	NM_205861.1
ILMN_1725726	DHRS2	0.014180417	down	NM_182908.3
ILMN_1733998	DHRS9	0.015453956	down	NM_005771.3
ILMN_1658356	DKFZP586H2123	0.032202482	down	NM_015430.2
ILMN_1773337	DKK1	1.40E-04	down	NM_012242.2
ILMN_1743373	DLL1	9.82E-04	down	NM_005618.3
ILMN_1778319	DMKN	0.01607549	down	NM_001035516.1
ILMN_1685052	DNAH1	0.049713753	down	NM_015512.3
ILMN_1776998	DNAJA4	4.56E-04	down	NM_018602.2
ILMN_1687683	DNAJC4	0.010037281	down	NM_005528.1
ILMN_1791679	DNER	3.36E-04	down	NM_139072.3
ILMN_1676128	DNMT3A	0.008798456	down	NM_175630.1
ILMN_1801226	DOCK6	0.024661653	down	NM_020812.1
ILMN_1692535	DPP4	0.001665569	down	NM_001935.3
ILMN_1712506	DPP6	0.03692527	down	NM_130797.2
ILMN_1673069	DPP9	9.71E-04	down	NM_139159.3
ILMN_1730201	DTNA	5.19E-04	down	NM_001392.3
ILMN_1798819	DTNB	0.030025776	down	NM_021907.3

ILMN_1653115	ECH1	0.023810888	down	NM_001398.2
ILMN_1767322	EDAR	1.06E-04	down	NM_022336.2
ILMN_1811616	EEPD1	0.017261356	down	NM_030636.2
ILMN_1768773	EGLN2	0.03283143	down	NM_053046.2
ILMN_1769201	ELF3	0.0487704	down	NM_004433.3
ILMN_1684699	ELF5	7.18E-04	down	NM_001422.2
ILMN_1813270	ELF5	8.21E-04	down	NM_198381.1
ILMN_1784320	ELMO1	0.043681003	down	NM_014800.9
ILMN_1798123	ELOVL1	0.012087655	down	NM_022821.2
ILMN_1801421	EMD	0.018889273	down	NM_000117.1
ILMN_1697268	EMILIN2	0.00149258	down	NM_032048.2
ILMN_1709593	EN1	0.002162563	down	NM_001426.3
ILMN_1710756	ENO1	0.03950261	down	NM_001428.2
ILMN_1765796	ENO2	0.033994094	down	NM_001975.2
ILMN_1675325	ENPEP	0.018972367	down	NM_001977.3
ILMN_1784967	EPB41L4B	0.002327866	down	NM_018424.2
ILMN_1671686	EPB49	0.015307781	down	NM_001978.1
ILMN_1675797	EPDR1	0.032991953	down	NM_017549.3
ILMN_1731001	ERICH1	0.018974869	down	NM_207332.1
ILMN_1700549	ERLIN2	0.002052658	down	NM_007175.5
ILMN_1744963	ERO1L	0.011302624	down	NM_014584.1
ILMN_1661994	ESRRG	8.20E-05	down	NM_206594.1
ILMN_1793267	ETHE1	0.021391267	down	NM_014297.3
ILMN_1803367	EVI1	0.031227868	down	NM_005241.1
ILMN_1718783	EXTL1	0.010803289	down	NM_004455.2
ILMN_1717163	F13A1	0.002538826	down	NM_000129.3
ILMN_1797009	F3	0.004581275	down	NM_001993.2
ILMN_1791531	FA2H	0.032598425	down	NM_024306.2
ILMN_1670134	FADS1	0.010711459	down	NM_013402.3
ILMN_1781536	FAH	0.019892905	down	NM_000137.1
ILMN_1745112	FAM102A	0.001732624	down	NM_001035254.1
ILMN_1790062	FAM105B	0.014451151	down	NM_138348.4
ILMN_1679641	FAM120B	0.027597925	down	NM_032448.1
ILMN_1737580	FAM152B	0.00294016	down	NM_015704.1
ILMN_1773780	FAM173A	0.016292036	down	NM_023933.1
ILMN_1652797	FAM174B	0.002628997	down	NM_207446.2
ILMN_1752249	FAM38A	0.0061945	down	NM_014745.1
ILMN_1720433	FAM3D	2.26E-04	down	NM_138805.2
ILMN_1758943	FAM5C	0.039869763	down	NM_199051.1
ILMN_1658861	FAM86B1	0.027356302	down	NM_001083537.1
ILMN_1778255	FARSA	0.014914026	down	NM_004461.2
ILMN_1728799	FBP1	0.010099354	down	NM_000507.2
ILMN_1804005	FBP2	0.04175629	down	NM_003837.2
ILMN_1697561	FBXL16	0.011414175	down	NM_153350.2
ILMN_1803211	FBXO2	0.014025323	down	NM_012168.4
ILMN_1662451	FCER2	0.033022556	down	NM_002002.3
ILMN_1660027	FCGR2B	0.012842847	down	NM_004001.3

ILMN_1804174	FCGR2B	0.015857473	down	XM_938851.1
ILMN_1705302	FCGRT	0.013644516	down	NM_004107.3
ILMN_1654571	FCHO1	0.004220631	down	NM_015122.1
ILMN_1804248	FDPS	0.038728707	down	NM_002004.2
ILMN_1662880	FIS	0.016426694	down	NM_175616.2
ILMN_1782045	FKBP4	0.019430088	down	NM_002014.2
ILMN_1709725	FLJ16165	0.033022054	down	NM_001004318.1
ILMN_1692464	FLJ20699	3.26E-04	down	NM_017931.1
ILMN_1711928	FLJ20920	0.011825501	down	NM_025149.3
ILMN_1751897	FLJ32569	0.004197324	down	XM_001132361.1
ILMN_1722609	FLJ32569	0.008052577	down	XM_001132361.1
ILMN_1768919	FLJ35880	8.74E-05	down	NM_153264.3
ILMN_1789048	FLJ90231	0.002696118	down	XM_001131501.1
ILMN_1684401	FMO1	0.039336164	down	NM_002021.1
ILMN_1732158	FMO2	0.01801342	down	NM_001460.2
ILMN_1811632	FMO5	0.049998805	down	NM_001461.1
ILMN_1761084	FNDC5	0.01600652	down	NM_153756.1
ILMN_1661733	FOLR1	8.90E-04	down	NM_016731.2
ILMN_1745963	FOLR2	4.09E-04	down	NM_000803.3
ILMN_1766650	FOXA1	1.71E-04	down	NM_004496.2
ILMN_1680973	FOXF1	0.014989023	down	NM_001451.2
ILMN_1681456	FOXL2	5.25E-07	down	NM_023067.2
ILMN_1714280	FOXL2	1.05E-04	down	XM_001131060.1
ILMN_1796851	FOXL2	0.007666925	down	XM_001131060.1
ILMN_1678961	FRMD4A	0.013814801	down	NM_018027.3
ILMN_1752728	FUCA1	0.008476541	down	NM_000147.3
ILMN_1794659	FUT2	4.29E-05	down	NM_001097638.1
ILMN_1665372	FXYD3	1.99E-04	down	NM_021910.1
ILMN_1791580	FXYD3	0.015540982	down	NM_005971.2
ILMN_1784706	GABRE	0.04640181	down	NM_004961.3
ILMN_1689146	GABRP	9.84E-04	down	NM_014211.1
ILMN_1694075	GADD45A	0.032835502	down	NM_001924.2
ILMN_1682015	GAL	0.031080427	down	NM_015973.3
ILMN_1792168	GALE	1.87E-05	down	NM_000403.3
ILMN_1737949	GALNS	0.009351644	down	NM_000512.3
ILMN_1735157	GALNT12	0.014843639	down	NM_024642.3
ILMN_1724059	GAS2L1	0.028971283	down	NM_152236.1
ILMN_1779558	GAS6	1.27E-05	down	NM_000820.1
ILMN_1784749	GAS6	0.001071924	down	NM_000820.1
ILMN_1674560	GBA2	0.007786376	down	NM_020944.2
ILMN_1652906	GBGT1	0.04655053	down	NM_021996.3
ILMN_1725311	GCGR	9.57E-05	down	NM_000160.2
ILMN_1712082	GCNT3	0.002681892	down	NM_004751.1
ILMN_1734153	GDI1	0.0397237	down	NM_001493.1
ILMN_1726549	GDPD2	6.02E-06	down	NM_017711.2
ILMN_1774901	GDPD3	0.010748497	down	NM_024307.2
ILMN_1701643	GDPD5	0.028681446	down	NM_030792.5

ILMN_1725471	GK	0.035018627	down	NM_000167.3
ILMN_1806754	GLDC	0.023525279	down	NM_000170.2
ILMN_1669631	GLRB	0.04385008	down	NM_000824.2
ILMN_1736742	GLT25D2	0.014592545	down	NM_015101.2
ILMN_1713756	GLUD1	0.02685491	down	NM_005271.1
ILMN_1653496	GLUL	0.026865084	down	NM_001033056.1
ILMN_1773645	GMPPB	0.011238354	down	NM_021971.1
ILMN_1730546	GNAO1	0.009516682	down	NM_020988.1
ILMN_1728107	GNG7	0.007503444	down	NM_052847.2
ILMN_1736757	GNPTAB	0.024605269	down	NM_024312.3
ILMN_1656145	GOT1	0.012721471	down	NM_002079.1
ILMN_1694106	GPD1L	0.006810485	down	NM_015141.2
ILMN_1677962	GPHN	0.010942975	down	NM_001024218.1
ILMN_1655348	GPR1	0.021474876	down	NM_001098199.1
ILMN_1703765	GPR12	5.63E-04	down	NM_005288.1
ILMN_1683492	GPR172B	0.049059987	down	NM_017986.2
ILMN_1701947	GPR34	0.016914109	down	NM_005300.3
ILMN_1703326	GPR44	0.009575901	down	NM_004778.2
ILMN_1671142	GPR68	0.003064467	down	NM_003485.3
ILMN_1724211	GPRC5C	7.84E-04	down	NM_022036.2
ILMN_1726666	GPX3	0.034647427	down	NM_002084.3
ILMN_1793832	GRB14	0.042692326	down	NM_004490.2
ILMN_1678816	GREM2	3.08E-06	down	NM_022469.3
ILMN_1725241	GSTK1	0.015484894	down	NM_015917.1
ILMN_1750790	GSTM5	0.006271533	down	NM_000851.2
ILMN_1758398	GUK1	0.005315908	down	NM_000858.4
ILMN_1711289	GYS1	0.020741021	down	NM_002103.3
ILMN_1719906	HADH	0.01174638	down	NM_005327.2
ILMN_1767474	HAO2	0.015561202	down	NM_016527.2
ILMN_1792323	HDC	3.55E-04	down	NM_002112.2
ILMN_1804150	HIBADH	0.019968385	down	NM_152740.2
ILMN_1656977	HIBCH	0.013434161	down	NM_198047.1
ILMN_1675936	HIGD1B	0.005667379	down	NM_016438.2
ILMN_1651496	HIST1H2BD	0.02501238	down	NM_138720.1
ILMN_1761733	HLA-DMB	0.0308621	down	NM_002118.3
ILMN_1680144	HLA-DQA2	0.044091154	down	NM_020056.2
ILMN_1792669	HLCS	0.03176872	down	NM_000411.4
ILMN_1657395	HMGCR	0.006367619	down	NM_000859.1
ILMN_1797728	HMGCS1	4.64E-04	down	NM_002130.6
ILMN_1682147	HOOK2	0.006580335	down	NM_013312.2
ILMN_1799836	HOXA2	7.45E-08	down	NM_006735.3
ILMN_1677018	HOXA4	3.59E-04	down	NM_002141.4
ILMN_1753613	HOXA5	4.02E-07	down	NM_019102.2
ILMN_1739582	HOXA9	4.38E-06	down	NM_152739.3
ILMN_1810274	HOXB2	5.38E-07	down	NM_002145.3
ILMN_1689874	HOXB3	5.54E-06	down	NM_002146.4
ILMN_1674908	HOXB5	1.22E-05	down	NM_002147.3

ILMN_1702125	HOXB7	3.64E-04	down	NM_004502.3
ILMN_1719975	HOXC4	4.70E-05	down	NM_014620.4
ILMN_1794492	HOXC6	3.03E-06	down	NM_004503.3
ILMN_1718285	HOXC8	6.71E-07	down	NM_022658.3
ILMN_1718898	HOXC9	8.20E-04	down	NM_006897.1
ILMN_1714691	HOXD10	3.04E-04	down	NM_002148.3
ILMN_1659792	HOXD9	0.003292323	down	NM_014213.2
ILMN_1764850	HPCAL1	0.010042178	down	NM_134421.1
ILMN_1655312	HPS5	0.001391987	down	NM_007216.3
ILMN_1807633	HRSP12	0.038709067	down	NM_005836.2
ILMN_1813350	HSD11B2	0.005176588	down	NM_000196.3
ILMN_1683844	HSD17B13	0.003376516	down	NM_178135.2
ILMN_1780693	HSD3B1	0.013786972	down	NM_000862.2
ILMN_1812669	HTRA3	0.024238175	down	NM_053044.2
ILMN_1704598	HYAL4	0.006240988	down	NM_012269.1
ILMN_1673649	HYOU1	0.04662294	down	NM_006389.2
ILMN_1814787	ICA1	6.20E-04	down	NM_004968.2
ILMN_1812567	ICA1	0.025351208	down	NM_022307.1
ILMN_1786823	ICAM2	0.037232164	down	NM_001099786.1
ILMN_1675671	ICOSLG	0.027601857	down	NM_015259.4
ILMN_1679299	IGSF1	1.18E-04	down	NM_001555.2
ILMN_1652825	IL10RA	0.002657185	down	NM_001558.2
ILMN_1665682	IL15RA	0.005702656	down	NM_002189.2
ILMN_1767523	IL17RB	0.006730394	down	NM_018725.3
ILMN_1781700	IL18R1	0.00308629	down	NM_003855.2
ILMN_1658483	IL1A	1.06E-04	down	NM_000575.3
ILMN_1697710	IL1F7	0.00208504	down	NM_014439.3
ILMN_1809099	IL33	0.006943567	down	NM_033439.2
ILMN_1730294	INO80C	4.79E-04	down	NM_194281.3
ILMN_1686989	INSIG1	0.001958023	down	NM_198336.1
ILMN_1793474	INSIG1	0.001104111	down	NM_198336.1
ILMN_1796968	INTS5	0.045282464	down	NM_030628.1
ILMN_1769433	IQGAP2	1.05E-06	down	NM_006633.2
ILMN_1745964	IRAK2	0.003611255	down	NM_001570.3
ILMN_1765649	IRF3	0.048093244	down	NM_001571.2
ILMN_1735432	ISCU	0.040702548	down	NM_014301.2
ILMN_1789505	ITPR1	0.004440782	down	NM_002222.4
ILMN_1692896	JMJD4	0.012328932	down	NM_023007.1
ILMN_1676247	JPH2	0.016903877	down	NM_175913.3
ILMN_1711092	KCNB1	0.002219713	down	NM_004975.2
ILMN_1744403	KCNIP3	0.001703636	down	NM_013434.4
ILMN_1709847	KCNJ13	0.01159884	down	NM_002242.2
ILMN_1735779	KCNJ8	0.023551013	down	NM_004982.2
ILMN_1766918	KCNK5	0.006270968	down	NM_003740.3
ILMN_1761903	KCNS1	1.71E-04	down	NM_002251.3
ILMN_1657495	KIAA0152	0.001223003	down	NM_014730.2
ILMN_1664960	KIAA0284	0.04237459	down	NM_015005.1

ILMN_1741398	KIAA0406	0.0271216	down	NM_014657.1
ILMN_1693233	KIAA0513	0.033125777	down	NM_014732.2
ILMN_1677968	KIAA0649	0.019235961	down	NM_014811.3
ILMN_1771482	KIAA1324	0.004451396	down	NM_020775.2
ILMN_1668619	KIAA1467	2.46E-06	down	NM_020853.1
ILMN_1790160	KIT	0.012244917	down	NM_001093772.1
ILMN_1801428	KRT77	0.004405615	down	NM_175078.1
ILMN_1753584	KRT8	0.006451551	down	NM_002273.2
ILMN_1795442	LAMA4	0.03419795	down	NM_002290.2
ILMN_1769782	LAX1	0.037485246	down	NM_017773.2
ILMN_1679960	LCE1A	0.005007254	down	NM_178348.1
ILMN_1718420	LCE1D	0.002600523	down	NM_178352.2
ILMN_1769684	LCE1E	0.001556945	down	NM_178353.1
ILMN_1716471	LCE1F	0.004832437	down	NM_178354.1
ILMN_1653282	LCE2A	0.004222743	down	NM_178428.3
ILMN_1795711	LCE2B	0.002122433	down	NM_014357.3
ILMN_1709708	LCE2C	0.048659917	down	NM_178429.2
ILMN_1656706	LCE2D	0.007320193	down	NM_178430.2
ILMN_1743296	LCE5A	7.38E-04	down	NM_178438.4
ILMN_1692223	LCN2	0.044391926	down	NM_005564.3
ILMN_1654890	LENG8	0.016486583	down	NM_052925.1
ILMN_1676265	LEPR	0.01675046	down	NM_001003679.1
ILMN_1731966	LEPR	0.003697327	down	NM_001003679.1
ILMN_1696391	LEPR	0.002922342	down	NM_002303.3
ILMN_1698019	LGMN	7.53E-04	down	NM_001008530.1
ILMN_1752199	LHPP	3.48E-04	down	NM_022126.2
ILMN_1732919	LILRB5	0.005684756	down	NM_006840.3
ILMN_1656886	LIN37	0.025396124	down	NM_019104.1
ILMN_1812700	LIPC	0.01513038	down	NM_000236.1
ILMN_1713841	LNX1	0.011021907	down	NM_032622.1
ILMN_1812867	LOC126767	0.010778109	down	XR_000192.3
ILMN_1712837	LOC148766	0.036020033	down	XM_086308.4
ILMN_1660519	LOC285382	0.043833613	down	NM_001025266.1
ILMN_1749834	LOC388588	0.001868366	down	XM_932890.2
ILMN_1713141	LOC389641	0.002694006	down	XM_944097.2
ILMN_1671292	LOC390705	0.014503969	down	XR_015850.1
ILMN_1713813	LOC400578	0.01459656	down	XR_017543.1
ILMN_1656925	LOC401180	0.015007321	down	XM_379325.2
ILMN_1664154	LOC440030	3.54E-04	down	XM_498510.2
ILMN_1791467	LOC440503	0.024871398	down	NM_001013706.1
ILMN_1683250	LOC440731	2.69E-05	down	XM_933693.2
ILMN_1663081	LOC440925	0.007377338	down	NM_001013712.1
ILMN_1811866	LOC642393	0.01417304	down	XM_930798.1
ILMN_1692045	LOC643100	0.013258868	down	XM_931316.1
ILMN_1668239	LOC643719	0.001589265	down	XM_944377.2
ILMN_1678553	LOC644222	0.022409922	down	XM_932052.2
ILMN_1673104	LOC644571	0.017572375	down	XM_927686.1

ILMN_1671971	LOC644743	0.015084573	down	XR_016703.1
ILMN_1661743	LOC644760	0.02741793	down	XM_932354.1
ILMN_1676712	LOC645553	0.008244811	down	XM_932965.2
ILMN_1694778	LOC646723	7.48E-04	down	XR_017241.1
ILMN_1692499	LOC647281	8.99E-04	down	XM_943346.2
ILMN_1693293	LOC648176	5.27E-04	down	XM_937221.1
ILMN_1741597	LOC650200	0.013296028	down	XM_939284.2
ILMN_1692517	LOC653381	0.007419966	down	XR_017364.1
ILMN_1800465	LOC654042	0.008991553	down	XM_945239.1
ILMN_1753340	LOC728226	0.031027379	down	XM_001128310.1
ILMN_1737387	LOC728441	0.03390394	down	XM_001129412.1
ILMN_1675542	LOC729148	0.030802947	down	XM_001129792.1
ILMN_1710410	LOC730413	0.005470058	down	XM_001123368.1
ILMN_1675482	LOC732425	0.015232227	down	XM_001133480.1
ILMN_1805225	LPCAT3	0.003132957	down	NM_005768.5
ILMN_1671554	LPIN1	0.027593171	down	NM_145693.1
ILMN_1809417	LRFN4	0.012606708	down	NM_024036.3
ILMN_1696587	LRP1B	0.004991886	down	NM_018557.2
ILMN_1732913	LRP6	0.04039502	down	NM_002336.2
ILMN_1755047	LRRC2	0.031397007	down	NM_024512.2
ILMN_1680757	LRRC26	2.02E-04	down	NM_001013653.2
ILMN_1746148	LRRC33	0.001478043	down	NM_198565.1
ILMN_1724266	LYPD2	0.019307159	down	NM_205545.1
ILMN_1808114	LYVE1	0.00480334	down	NM_006691.3
ILMN_1740960	MACROD1	0.001332555	down	NM_014067.2
ILMN_1763884	Magmas	0.004887189	down	NM_016069.8
ILMN_1679391	MAMDC2	0.01138348	down	NM_153267.3
ILMN_1740759	MAMDC4	0.008171935	down	NM_206920.2
ILMN_1800733	MANBA	0.049571287	down	NM_005908.3
ILMN_1727360	MAOB	0.00246842	down	NM_000898.3
ILMN_1694240	MAP2K1	0.048192132	down	NM_002755.2
ILMN_1673896	MAP3K13	0.022593146	down	NM_004721.3
ILMN_1694539	MAP3K6	0.038958088	down	NM_004672.3
ILMN_1774132	MAP6	0.007515299	down	NM_033063.1
ILMN_1756352	MAPBPIP	0.034637462	down	NM_014017.1
ILMN_1662126	MATN4	0.001907326	down	NM_030592.1
ILMN_1666109	MB	6.78E-04	down	NM_005368.2
ILMN_1764383	MCOLN1	0.021257276	down	NM_020533.1
ILMN_1775962	MCOLN3	0.005743013	down	NM_018298.9
ILMN_1770610	MERTK	0.023995616	down	NM_006343.2
ILMN_1806603	MESP1	0.004018124	down	NM_018670.2
ILMN_1688775	METRNL	0.035949133	down	XM_941466.2
ILMN_1761912	MGAT1	0.001554511	down	NM_002406.2
ILMN_1853824	MGAT3	0.013205485	down	NM_002409.4
ILMN_1769601	MGC16169	0.021262156	down	NM_033115.2
ILMN_1663575	MGC87042	0.046487622	down	XM_001128032.1
ILMN_1803005	MMACHC	0.034109265	down	NM_015506.2

ILMN_1718646	MMP15	0.04081503	down	NM_002428.2
ILMN_1679694	MMP27	0.019430988	down	NM_022122.2
ILMN_1791508	MMP28	0.016101653	down	NM_024302.3
ILMN_1685403	MMP7	3.35E-04	down	NM_002423.3
ILMN_1725700	MOV10	0.023300515	down	NM_020963.2
ILMN_1761262	MPI	0.003823537	down	NM_002435.1
ILMN_1733675	MPP1	0.012650639	down	NM_002436.2
ILMN_1705676	MRAP	0.013834188	down	NM_178817.3
ILMN_1794589	MRGPRX2	0.029502992	down	NM_054030.2
ILMN_1763264	MRPL2	0.044476572	down	NM_015950.3
ILMN_1783681	MRPL34	0.043736335	down	NM_023937.2
ILMN_1813682	MRPL53	0.012848871	down	NM_053050.2
ILMN_1815043	MRPS2	0.011971599	down	NM_016034.2
ILMN_1760441	MRPS5	0.008912083	down	NM_031902.3
ILMN_1741712	MS4A4A	0.013059248	down	NM_024021.2
ILMN_1688154	MST1R	0.008960963	down	NM_002447.2
ILMN_1785324	MTHFD1	0.001017689	down	NM_005956.2
ILMN_1677314	MUC1	0.006234659	down	NM_002456.4
ILMN_1756992	MUC1	0.001514683	down	NM_001044391.1
ILMN_1798931	MUC7	0.02633402	down	NM_152291.1
ILMN_1657550	MVD	0.005730209	down	NM_002461.1
ILMN_1752075	MYBPC1	4.88E-06	down	NM_206819.1
ILMN_1795119	MYH3	0.04144656	down	NM_002470.2
ILMN_1805999	MYO1D	7.04E-04	down	NM_015194.1
ILMN_1808789	MYO5C	0.004696057	down	NM_018728.2
ILMN_1811719	MYO7A	6.90E-04	down	NM_000260.2
ILMN_1686392	NAG	0.001348302	down	NM_015909.2
ILMN_1809490	NAP5	7.29E-05	down	NM_207363.2
ILMN_1703374	NAV1	0.041859243	down	NM_020443.2
ILMN_1683441	NCAPD3	0.001338411	down	NM_015261.2
ILMN_1687316	NCDN	0.006260583	down	NM_014284.2
ILMN_1784641	NDUFA3	0.020433716	down	NM_004542.2
ILMN_1749738	NECAB3	0.011415896	down	NM_031231.3
ILMN_1733627	NEDD4L	0.002221941	down	NM_015277.3
ILMN_1812622	NEIL1	0.021725878	down	NM_024608.1
ILMN_1744959	NFX1	0.004526704	down	NM_002504.3
ILMN_1795025	NFX1	0.015362252	down	NM_147133.1
ILMN_1807211	NICN1	0.021134948	down	NM_032316.3
ILMN_1731206	NKD2	0.013701841	down	NM_033120.2
ILMN_1811363	NOVA1	0.011385872	down	NM_006491.2
ILMN_1684210	NPAL3	0.041297715	down	NM_020448.3
ILMN_1739690	NPFFR2	8.86E-04	down	NM_004885.1
ILMN_1685608	NPTX2	1.95E-04	down	NM_002523.1
ILMN_1687392	NRK	0.030120669	down	NM_198465.2
ILMN_1692163	NSDHL	0.017825976	down	NM_015922.1
ILMN_1697962	NSMCE1	0.035913553	down	NM_145080.3
ILMN_1751958	NSUN5	0.027097408	down	NM_148956.1

ILMN_1714067	NTRK2	0.003310891	down	NM_001007097.1
ILMN_1711314	NUDT5	0.016850345	down	NM_014142.2
ILMN_1721540	NWD1	0.010135769	down	NM_001007525.1
ILMN_1741214	NXPH4	0.03751713	down	XM_938935.2
ILMN_1733869	OGDH	8.23E-04	down	NM_002541.2
ILMN_1674696	OLAH	0.007290253	down	NM_018324.1
ILMN_1670079	OMA1	0.007052742	down	NM_145243.3
ILMN_1838320	ONECUT2	0.037886064	down	NM_004852.2
ILMN_1711030	OPLAH	0.019516775	down	NM_017570.2
ILMN_1738539	OPLAH	0.04751055	down	XM_001129650.1
ILMN_1670844	OPN3	0.014272459	down	NM_014322.2
ILMN_1716988	OPN3	0.045023024	down	NM_014322.2
ILMN_1709091	OXGR1	0.003998722	down	NM_080818.3
ILMN_1660031	P2RY6	0.049180277	down	NM_176796.1
ILMN_1747442	P4HA1	0.018054988	down	NM_001017962.1
ILMN_1682919	PAFAH2	0.001773542	down	NM_000437.3
ILMN_1708223	PAK6	0.003774616	down	NM_020168.3
ILMN_1783497	PANK1	0.046047017	down	NM_148978.1
ILMN_1667592	PARVB	0.013559008	down	NM_001003828.1
ILMN_1787919	PARVB	6.64E-04	down	NM_013327.3
ILMN_1795906	PCBD1	0.022509372	down	NM_000281.2
ILMN_1786105	PCBD1	0.001990153	down	NM_000281.2
ILMN_1761010	PCCB	0.04266059	down	NM_000532.3
ILMN_1673129	PCDH12	0.009080071	down	NM_016580.2
ILMN_1746888	PCOLCE2	0.007722486	down	NM_013363.2
ILMN_1699545	PCSK7	0.022245737	down	NM_004716.2
ILMN_1802257	PCTP	0.025334168	down	NM_021213.1
ILMN_1652846	PCYT2	0.001071277	down	NM_002861.2
ILMN_1681356	PDE2A	0.008535544	down	NM_002599.1
ILMN_1803094	PDGFD	0.00202461	down	NM_033135.3
ILMN_1652357	PDHX	0.03278074	down	NM_003477.1
ILMN_1695299	PDLIM3	0.00140172	down	NM_014476.1
ILMN_1670490	PDPN	0.015937839	down	NM_001006625.1
ILMN_1672504	PDXK	0.04137996	down	NM_003681.4
ILMN_1708916	PDZD4	0.038563352	down	NM_032512.2
ILMN_1708580	PDZK1IP1	2.70E-04	down	NM_005764.3
ILMN_1814120	PECR	0.03941429	down	NM_018441.3
ILMN_1717674	PEPD	0.031237263	down	NM_000285.2
ILMN_1753010	PET112L	0.004147905	down	NM_004564.1
ILMN_1660847	PFKFB3	0.04811277	down	NM_004566.2
ILMN_1653292	PFKFB4	0.031846333	down	NM_004567.2
ILMN_1800659	PGM1	0.001320979	down	NM_002633.2
ILMN_1836744	PGP	0.005175894	down	NM_001042371.2
ILMN_1746968	PHF1	0.006692253	down	NM_024165.1
ILMN_1736015	PHF17	0.02659249	down	NM_024900.3
ILMN_1671557	PHLDA2	0.03635441	down	NM_003311.3
ILMN_1814661	PHLPP	0.001311326	down	NM_194449.1

ILMN_1676611	PHPT1	0.0343497	down	NM_014172.3
ILMN_1661895	PI15	1.92E-04	down	NM_015886.3
ILMN_1693192	PI3	3.89E-04	down	NM_002638.2
ILMN_1691291	PIGS	0.014082406	down	NM_033198.2
ILMN_1738759	PIGT	0.00924072	down	NM_015937.3
ILMN_1701213	PIP4K2B	0.03548092	down	NM_003559.4
ILMN_1742788	PKD1L2	0.003891726	down	NM_052892.3
ILMN_1717886	PKHD1L1	0.045283455	down	NM_177531.4
ILMN_1797184	PKN3	0.027095534	down	NM_013355.3
ILMN_1756910	PLA2G15	0.002534989	down	NM_012320.3
ILMN_1740586	PLA2G2A	0.001051057	down	NM_000300.2
ILMN_1701195	PLA2G7	0.006677352	down	NM_005084.2
ILMN_1683016	PLA2R1	0.00468682	down	NM_001007267.1
ILMN_1704424	PLA2R1	0.002412436	down	NM_001007267.1
ILMN_1815121	PLAGL1	0.03964413	down	NM_001080951.1
ILMN_1747451	PLCXD1	0.01723415	down	NM_018390.2
ILMN_1797557	PLEKHA6	0.006187835	down	NM_014935.2
ILMN_1783231	PLEKHB1	0.036781274	down	NM_021200.1
ILMN_1657836	PLEKHG2	0.046379346	down	NM_022835.1
ILMN_1744614	PLEKHG6	0.012161595	down	NM_018173.2
ILMN_1699254	PLEKHH1	0.032949198	down	NM_020715.2
ILMN_1787673	PLLP	0.002762764	down	NM_015993.1
ILMN_1684391	PLOD1	0.018212726	down	NM_000302.2
ILMN_1656132	PNLDC1	0.007069864	down	NM_173516.1
ILMN_1684289	PNPO	0.015948566	down	NM_018129.2
ILMN_1670037	POLR2L	0.00727187	down	NM_021128.3
ILMN_1677768	POR	0.004839461	down	NM_000941.2
ILMN_1805827	PPA1	0.005406993	down	NM_021129.3
ILMN_1750062	PPARGC1A	3.14E-04	down	NM_013261.3
ILMN_1713846	PPM1H	0.023786966	down	NM_020700.1
ILMN_1781198	PPP1R3D	9.99E-05	down	NM_006242.3
ILMN_1709247	PPP2R1B	0.014425299	down	NM_002716.4
ILMN_1750664	PPT2	0.005867308	down	NM_138717.1
ILMN_1662989	PRB2	0.007670424	down	NM_006248.1
ILMN_1779825	PRDM2	0.03973659	down	NM_012231.3
ILMN_1656942	PRICKLE3	0.02953502	down	NM_006150.3
ILMN_1671365	PRODH	4.69E-04	down	NM_016335.2
ILMN_1786720	PROM1	0.001442107	down	NM_006017.1
ILMN_1736154	ProSAPiP1	0.039389953	down	NM_014731.2
ILMN_1753665	PRR4	0.027663829	down	NM_001098538.1
ILMN_1713829	PTGES	0.005770266	down	NM_004878.3
ILMN_1667692	PTGIS	0.034771126	down	NM_000961.3
ILMN_1729075	PTH2R	0.001336782	down	NM_005048.2
ILMN_1760575	PTP4A1	0.04130545	down	NM_003463.3
ILMN_1698885	PTPRT	5.10E-06	down	NM_007050.5
ILMN_1720322	PTS	0.034657095	down	NM_000317.1
ILMN_1789384	QSOX2	0.001357476	down	NM_181701.3

ILMN_1790317	RAB26	0.01815551	down	NM_014353.4
ILMN_1751571	RAD23A	0.004016139	down	NM_005053.2
ILMN_1776519	RAP1GAP	0.012652201	down	NM_002885.1
ILMN_1769412	RAPGEF1	0.008702513	down	NM_198679.1
ILMN_1654586	RASA3	6.89E-04	down	NM_007368.2
ILMN_1793517	RASAL1	0.005644111	down	NM_004658.1
ILMN_1727045	RASGRP3	0.033329207	down	NM_170672.1
ILMN_1745820	RASSF6	0.00252176	down	NM_201431.1
ILMN_1689625	RAX	0.006108642	down	NM_013435.2
ILMN_1688630	RECK	0.024828898	down	NM_021111.1
ILMN_1800787	RFTN1	0.015432269	down	NM_015150.1
ILMN_1668559	RGS10	0.03222846	down	NM_001005339.1
ILMN_1696828	RGS14	0.012841324	down	NM_006480.4
ILMN_1675172	RGS9BP	0.03626322	down	NM_207391.2
ILMN_1753143	RHPN2	2.18E-04	down	NM_033103.3
ILMN_1660858	RIN1	0.010839044	down	NM_004292.2
ILMN_1737847	RMND5B	0.034867585	down	NM_022762.3
ILMN_1712849	RNASE7	0.014764107	down	NM_032572.2
ILMN_1787259	RNF212	0.006620213	down	NM_194439.1
ILMN_1668766	ROPN1	0.040949475	down	NM_017578.2
ILMN_1781758	ROPN1B	0.045226295	down	NM_001012337.1
ILMN_1811234	ROS1	6.06E-04	down	NM_002944.2
ILMN_1693717	RPH3AL	0.001186401	down	NM_006987.2
ILMN_1729033	RPL9	0.04166131	down	NM_001024921.2
ILMN_1657515	RPS6KA5	0.030662265	down	NM_004755.2
ILMN_1753439	RPTN	4.85E-07	down	XM_937200.2
ILMN_1699772	RRAGD	0.02723581	down	NM_021244.3
ILMN_1682937	RSPO1	7.75E-06	down	NM_001038633.2
ILMN_1756928	RTN1	0.006439577	down	NM_021136.2
ILMN_1749115	RTN2	0.04738847	down	NM_206901.1
ILMN_1787461	RUNX3	1.00E-04	down	NM_004350.2
ILMN_1801216	S100P	0.005177937	down	NM_005980.2
ILMN_1701017	SAA1	0.025325602	down	NM_000331.3
ILMN_1767801	SAMD10	0.012488452	down	NM_080621.4
ILMN_1794085	SAPS1	0.028830832	down	NM_014931.3
ILMN_1753342	SAT1	0.036551565	down	NM_002970.1
ILMN_1738657	SATB2	0.006106715	down	NM_015265.1
ILMN_1808811	SBNO2	0.044066858	down	NM_014963.2
ILMN_1720889	SC4MOL	0.00286216	down	NM_001017369.1
ILMN_1800843	SCAMP4	0.032947432	down	NM_079834.2
ILMN_1751062	SCARA5	3.55E-05	down	NM_173833.4
ILMN_1714536	SCGB1D2	5.34E-05	down	NM_006551.3
ILMN_1732398	SCGB2A1	6.51E-05	down	NM_002407.1
ILMN_1723333	SCGB2A2	2.45E-04	down	NM_002411.2
ILMN_1679666	SCGB3A1	0.030099848	down	NM_052863.2
ILMN_1740917	SCNN1B	0.00139999	down	NM_000336.2
ILMN_1814215	SCNN1G	0.040091157	down	NM_001039.3

ILMN_1756439	SCRN1	9.23E-04	down	NM_014766.3
ILMN_1772537	SCTR	0.001739489	down	NM_002980.2
ILMN_1705107	SDCBP2	0.010014898	down	NM_015685.4
ILMN_1750674	SDSL	0.007937287	down	NM_138432.2
ILMN_1657483	SEC23B	1.64E-04	down	NM_032985.4
ILMN_1696133	SELI	0.030338246	down	NM_033505.2
ILMN_1769787	SELO	0.004274803	down	NM_031454.1
ILMN_1787680	SELS	0.03030125	down	NM_018445.4
ILMN_1765641	SEMA3A	0.021936854	down	NM_006080.2
ILMN_1651547	SEMA3E	0.02480911	down	NM_012431.1
ILMN_1657409	SERHL	0.016923679	down	NM_170694.1
ILMN_1712400	SERPINB6	0.01659352	down	NM_004568.4
ILMN_1767685	SERPINB7	0.026790721	down	NM_003784.2
ILMN_1724504	SETD3	0.038210634	down	NM_032233.2
ILMN_1778836	SFRS7	5.54E-04	down	NM_001031684.1
ILMN_1752046	SH2B3	0.008780085	down	NM_005475.1
ILMN_1772466	SH2D3A	0.02194706	down	NM_005490.1
ILMN_1762764	SH3BGRL2	0.009820405	down	NM_031469.2
ILMN_1653133	SH3D19	0.010854079	down	NM_001009555.2
ILMN_1756595	SH3TC1	0.005163032	down	NM_018986.3
ILMN_1721022	SHC1	0.007840182	down	NM_003029.3
ILMN_1807050	SHC4	0.018280204	down	NM_203349.2
ILMN_1811933	SHMT1	0.001908737	down	NM_004169.3
ILMN_1795118	SIDT1	0.001069193	down	NM_017699.2
ILMN_1678729	SIL1	0.034525923	down	NM_001037633.1
ILMN_1788315	SIN3B	0.004500377	down	NM_015260.1
ILMN_1799598	SIRT5	0.013928114	down	NM_012241.2
ILMN_1683059	SIRT5	0.040236086	down	NM_012241.2
ILMN_1657129	SKAP2	2.87E-05	down	NM_003930.3
ILMN_1720996	SLC12A2	0.00133285	down	NM_001046.2
ILMN_1805561	SLC14A1	0.006153208	down	NM_015865.2
ILMN_1716359	SLC19A3	0.03834284	down	NM_025243.3
ILMN_1738552	SLC1A3	0.00990769	down	NM_004172.3
ILMN_1707720	SLC1A5	7.16E-04	down	NM_005628.1
ILMN_1691048	SLC22A18AS	0.024764037	down	NM_007105.1
ILMN_1664168	SLC25A11	0.045607165	down	NM_003562.3
ILMN_1754864	SLC25A18	0.016634325	down	NM_031481.1
ILMN_1741392	SLC25A20	0.043082073	down	NM_000387.3
ILMN_1697544	SLC25A29	0.039351385	down	NM_001039355.1
ILMN_1760087	SLC26A3	0.026801972	down	NM_000111.1
ILMN_1717326	SLC29A3	0.024002781	down	NM_018344.3
ILMN_1778321	SLC2A6	0.015415433	down	NM_017585.2
ILMN_1687495	SLC37A1	0.002626453	down	NM_018964.3
ILMN_1656186	SLC41A1	0.004106274	down	NM_173854.4
ILMN_1730977	SLC44A4	0.008732732	down	NM_025257.2
ILMN_1735156	SLC4A11	1.83E-04	down	NM_032034.2
ILMN_1744191	SLC6A1	0.04528648	down	NM_003042.2

ILMN_1723287	SLC6A16	8.42E-04	down	NM_014037.2
ILMN_1806349	SLC6A8	0.03696519	down	NM_005629.1
ILMN_1774229	SLC7A4	0.017141404	down	NM_004173.2
ILMN_1738849	SLC9A2	0.00138364	down	NM_003048.3
ILMN_1758315	SLC9A9	0.014515182	down	NM_173653.1
ILMN_1752520	SLFN11	0.0049277	down	NM_152270.2
ILMN_1676449	SLIT2	0.040906753	down	NM_004787.1
ILMN_1802316	SMPD3	0.00979955	down	NM_018667.2
ILMN_1701933	SNCA	0.001390071	down	NM_007308.1
ILMN_1766165	SNCA	5.09E-04	down	NM_000345.2
ILMN_1668714	SNF1LK2	0.037762456	down	NM_015191.1
ILMN_1699100	SOAT1	0.04172994	down	NM_003101.4
ILMN_1789244	SOX8	0.005201588	down	NM_014587.2
ILMN_1787266	SPINK1	0.004282134	down	NM_003122.2
ILMN_1800739	SPINT2	0.005320218	down	NM_021102.2
ILMN_1703284	SPIRE2	0.018725425	down	NM_032451.1
ILMN_1676099	SPON2	0.03146569	down	NM_012445.1
ILMN_1705849	SPR	0.001574063	down	NM_003124.3
ILMN_1795359	SPRR2A	0.004153787	down	NM_005988.2
ILMN_1806059	SPRR2B	0.03802265	down	NM_001017418.1
ILMN_1729868	SPRYD4	0.012292002	down	NM_207344.2
ILMN_1793241	SRD5A1	0.003548417	down	NM_001047.2
ILMN_1781280	SRD5A2L2	2.13E-05	down	NM_001010874.3
ILMN_1759549	SRGAP2	0.027172353	down	NM_015326.2
ILMN_1709486	SRPX	0.024592279	down	NM_006307.3
ILMN_1676213	SRPX2	0.002363337	down	NM_014467.2
ILMN_1683313	ST3GAL1	0.020080525	down	NM_003033.2
ILMN_1699472	ST3GAL3	0.041541524	down	NM_174967.1
ILMN_1713496	ST3GAL5	0.024915077	down	NM_001042437.1
ILMN_1756501	ST6GAL1	0.01816951	down	NM_003032.2
ILMN_1687857	ST6GALNAC4	0.003034419	down	NM_175039.3
ILMN_1664859	ST8SIA1	0.011182939	down	NM_003034.2
ILMN_1718295	STAC2	8.49E-04	down	NM_198993.2
ILMN_1739253	STAMBP	2.19E-04	down	NM_006463.3
ILMN_1660451	STARD13	0.003288729	down	NM_178008.1
ILMN_1784364	STARD5	4.21E-04	down	NM_181900.2
ILMN_1733094	STEAP1	0.018798992	down	NM_012449.2
ILMN_1651692	STK10	0.004794037	down	NM_005990.3
ILMN_1791328	STK39	0.04492926	down	NM_013233.2
ILMN_1795679	STMN2	0.0352923	down	NM_007029.2
ILMN_1773901	STX12	0.008885363	down	NM_177424.2
ILMN_1803745	SUOX	0.008677946	down	NM_000456.2
ILMN_1709750	SUSD1	0.004451236	down	NM_022486.3
ILMN_1732189	SYCE1	4.48E-04	down	NM_130784.1
ILMN_1757081	SYN2	0.016960382	down	NM_003178.4
ILMN_1777362	SYT15	0.03036343	down	NM_031912.3
ILMN_1657760	SYT17	0.003152554	down	NM_016524.2

ILMN_1720623	SYTL3	0.005749659	down	NM_001009991.2
ILMN_1701487	TAOK2	0.036790676	down	NM_016151.2
ILMN_1707791	TAZ	0.007529782	down	NM_181311.1
ILMN_1701604	TBC1D8B	0.02232258	down	NM_017752.2
ILMN_1713449	TBX3	1.71E-04	down	NM_005996.3
ILMN_1734596	TC2N	0.006614189	down	NM_152332.3
ILMN_1740572	TCN2	0.014668781	down	NM_000355.2
ILMN_1751630	TDRD10	9.52E-05	down	NM_182499.3
ILMN_1685042	TENC1	0.04688194	down	NM_015319.2
ILMN_1750181	TESC	0.003725515	down	NM_017899.2
ILMN_1781623	TEX264	0.026769418	down	NM_015926.3
ILMN_1790595	TFAP2E	0.030191597	down	NM_178548.2
ILMN_1814657	TFAP4	0.034816664	down	NM_003223.1
ILMN_1736527	TFCP2L1	7.51E-04	down	NM_014553.1
ILMN_1709451	TFPT	0.03723658	down	NM_013342.2
ILMN_1726245	TGFBR2	0.011287666	down	NM_001024847.2
ILMN_1784287	TGFBR3	0.04712622	down	NM_003243.2
ILMN_1678842	THBS2	0.044186	down	NM_003247.2
ILMN_1794844	THRSP	0.03743322	down	NM_003251.2
ILMN_1815079	TICAM1	0.017705113	down	NM_182919.1
ILMN_1813260	TIMM17B	0.039626583	down	NM_005834.1
ILMN_1663399	TIMP4	0.007646821	down	NM_003256.2
ILMN_1659610	TJP3	3.00E-04	down	NM_014428.1
ILMN_1784655	TLCD1	0.03516647	down	NM_138463.2
ILMN_1661299	TLE6	0.006254032	down	NM_024760.1
ILMN_1690040	TM7SF2	0.038395718	down	NM_003273.2
ILMN_1693311	TMBIM6	0.009078092	down	NM_003217.2
ILMN_1803219	TMC4	0.007171284	down	NM_144686.1
ILMN_1781276	TMCO4	0.027486077	down	NM_181719.3
ILMN_1685709	TMEM125	0.001543108	down	NM_144626.1
ILMN_1700202	TMEM135	0.03320617	down	NM_022918.2
ILMN_1683470	TMEM139	0.003356544	down	NM_153345.1
ILMN_1791511	TMEM176A	0.04038032	down	NM_018487.2
ILMN_1808566	TMEM180	0.04877151	down	NM_024789.3
ILMN_1710078	TMEM181	0.0273013	down	NM_020823.1
ILMN_1739126	TMEM213	4.86E-04	down	NM_001085429.1
ILMN_1668246	TMEM52	0.002611809	down	NM_178545.2
ILMN_1782429	TMEM56	0.026113678	down	NM_152487.1
ILMN_1736911	TMOD1	0.04671401	down	NM_003275.2
ILMN_1791123	TMPRSS2	2.64E-04	down	NM_005656.2
ILMN_1785732	TNFAIP6	0.002244102	down	NM_007115.2
ILMN_1809842	TNNC1	0.002888099	down	NM_003280.1
ILMN_1664071	TNNT2	0.001934349	down	NM_000364.2
ILMN_1716370	TNS4	0.037570026	down	NM_032865.4
ILMN_1655068	TOM1L2	0.029533725	down	NM_001082968.1
ILMN_1711109	TOM1L2	0.009457503	down	NM_001082968.1
ILMN_1737805	TPCN1	0.031161942	down	NM_017901.3

ILMN_1714383	TPD52L1	0.001763337	down	NM_001003396.1
ILMN_1804629	TPK1	0.022487924	down	NM_001042482.1
ILMN_1769219	TPSG1	0.007491686	down	NM_012467.2
ILMN_1657509	TSEN54	0.025691694	down	NM_207346.2
ILMN_1655611	TSHZ2	1.63E-04	down	NM_173485.4
ILMN_1669881	TSPAN13	0.015262893	down	NM_014399.3
ILMN_1663113	TTLL12	0.021415832	down	NM_015140.2
ILMN_1702821	TTLL7	0.041621648	down	NM_024686.4
ILMN_1695829	TTYH2	0.010114613	down	NM_032646.5
ILMN_1775074	TUBGCP2	0.048374444	down	NM_006659.2
ILMN_1742730	TWIST2	0.03470155	down	XM_941980.1
ILMN_1799367	TXNDC14	0.028270112	down	NM_015959.1
ILMN_1699226	UBR4	6.39E-06	down	NM_020765.2
ILMN_1758816	UGT8	6.49E-04	down	NM_003360.2
ILMN_1774265	UNQ830	5.01E-04	down	NM_206895.1
ILMN_1664243	USE1	0.008160718	down	NM_018467.3
ILMN_1700024	UST	0.022305885	down	NM_005715.1
ILMN_1803882	VEGFA	0.001018716	down	NM_001025367.1
ILMN_1782352	VENTX	0.016800404	down	NM_014468.2
ILMN_1709434	VIT	2.34E-04	down	NM_053276.2
ILMN_1736751	VPS26B	0.015719676	down	NM_052875.3
ILMN_1666536	VSIG2	0.012845992	down	NM_014312.3
ILMN_1669409	VSIG4	1.80E-04	down	NM_007268.2
ILMN_1810797	WASF3	0.03892407	down	NM_006646.4
ILMN_1763196	WDR72	2.10E-04	down	NM_182758.1
ILMN_1689932	WDR85	0.024467146	down	NM_138778.2
ILMN_1744923	WFDC5	0.014075573	down	NM_145652.2
ILMN_1812461	WISP2	0.027166959	down	NM_003881.2
ILMN_1761684	WNK2	0.019883918	down	NM_006648.3
ILMN_1772824	WNT5B	6.54E-04	down	NM_032642.2
ILMN_1658619	WWC1	0.038713843	down	NM_015238.1
ILMN_1771057	XAB2	0.019048482	down	NM_020196.2
ILMN_1794349	XYLB	0.010615191	down	NM_005108.2
ILMN_1755354	YBX2	0.009380867	down	NM_015982.2
ILMN_1786852	ZCCHC3	0.031148305	down	NM_033089.6
ILMN_1655545	ZMYND12	0.004718081	down	NM_032257.3
ILMN_1763208	ZNF10	0.027285911	down	NM_015394.4
ILMN_1683487	ZNF154	0.006959617	down	NM_003444.1
ILMN_1711199	ZNF331	0.018150194	down	NM_001079907.1
ILMN_1710268	ZNF385D	0.001161134	down	NM_024697.1
ILMN_1657087	ZNF533	0.035981867	down	NM_152520.3
ILMN_1772155	ZNF536	0.001168757	down	NM_014717.1
ILMN_1742923	ZNF584	0.037255023	down	NM_173548.1
ILMN_1794823	ZNF626	0.003245883	down	NM_145297.3
ILMN_1713454	ZNF671	0.022794563	down	NM_024833.1
ILMN_1804263	ZNF76	0.015552781	down	NM_003427.3
ILMN_1715718	ZNF784	0.0248496	down	NM_203374.1

ILMN_1816244		0.020432008	down	BX647087
ILMN_1818256		0.006806329	down	BX103639
ILMN_1827736		0.004175909	down	BC038512
ILMN_1829555		0.002181943	down	BG210316
ILMN_1831106		2.83E-04	down	XM_378360
ILMN_1833727		7.27E-04	down	BX093803
ILMN_1833858		0.00358841	down	AF038185
ILMN_1835234		0.0071913	down	BX088859
ILMN_1837102		0.038903948	down	AK123875
ILMN_1843198		0.008707942	down	AK026966
ILMN_1846001		0.008578028	down	XM_379190
ILMN_1847047		0.030714454	down	BX106738
ILMN_1854270		0.002081577	down	BX109986
ILMN_1854765		3.00E-04	down	AK130706
ILMN_1859127		0.022296863	down	XM_373666
ILMN_1860638		0.015340978	down	BX537506
ILMN_1861270		0.002307866	down	BX538295
ILMN_1862521		0.017101318	down	AK093982
ILMN_1864166		0.01788501	down	BC080552
ILMN_1868655		0.024873398	down	AI916641
ILMN_1876470		0.008408183	down	AA935421
ILMN_1877721		0.009740097	down	BI034219
ILMN_1879059		0.003592594	down	AW452729
ILMN_1881909		0.010125764	down	BU536065
ILMN_1881960		0.001353629	down	CR622072
ILMN_1882311		0.03095526	down	DA529130
ILMN_1882590		0.04670105	down	AK094914
ILMN_1882652		0.046049245	down	BG697942
ILMN_1886422		0.04766479	down	BM702734
ILMN_1886493		0.0475192	down	AK026373
ILMN_1887267		1.80E-06	down	BX355045
ILMN_1887357		8.14E-05	down	AK095715
ILMN_1888609		0.028248243	down	BG120325
ILMN_1888901		0.012616899	down	BX106902
ILMN_1893555		2.06E-04	down	BX482494
ILMN_1897310		0.002075621	down	AI821112
ILMN_1902658		0.006080138	down	AK130294
ILMN_1904054		1.15E-04	down	AK123741
ILMN_1907042		0.035171557	down	AK123264
ILMN_1912997		0.022430291	down	AK123107
ILMN_1913060		0.005539166	down	AK126405
ILMN_1788778	11-Sep	4.07E-05	up	NM_018243.2
ILMN_1739594	ACOT11	0.01331739	up	NM_147161.2
ILMN_1760490	ACVR1	4.60E-04	up	NM_001105.2
ILMN_1727524	ADAM9	0.022963002	up	NM_003816.2
ILMN_1697628	ADARB1	0.038211305	up	NM_001112.2
ILMN_1679797	ADARB1	8.42E-04	up	NM_001112.2

ILMN_1666019	ADNP	0.027357435	up	NM_015339.2
ILMN_1802631	AGA	0.039686088	up	NM_000027.2
ILMN_1657862	AHCY	0.030541336	up	NM_000687.1
ILMN_1709348	ALDH1A1	1.09E-04	up	NM_000689.3
ILMN_1793859	ALDH2	0.022839986	up	NM_000690.2
ILMN_1702503	ALDH3A1	0.038672753	up	NM_000691.3
ILMN_1660856	ALG11	0.009669987	up	NM_001004127.1
ILMN_1779190	ALKBH8	0.028942138	up	NM_138775.1
ILMN_1713927	AMTN	7.97E-05	up	NM_212557.2
ILMN_1772612	ANGPTL2	0.001441415	up	NM_012098.2
ILMN_1813361	ANGPTL7	0.019938184	up	NM_021146.2
ILMN_1787064	ANKRD17	0.03131375	up	NM_032217.3
ILMN_1780894	ANTXR1	2.18E-04	up	NM_032208.1
ILMN_1670379	ANTXR1	0.008944513	up	NM_032208.1
ILMN_1689431	APCDD1L	2.19E-04	up	NM_153360.1
ILMN_1775441	ARHGAP24	0.042475104	up	NM_031305.2
ILMN_1796772	ARHGAP28	0.04239802	up	NM_001010000.1
ILMN_1797753	ARHGAP28	0.04891996	up	NM_030672.2
ILMN_1777691	ARL9	0.024376402	up	NM_206919.1
ILMN_1741156	ARMCX5	0.043102976	up	NM_022838.2
ILMN_1716026	ARMCX6	0.001899161	up	NM_019007.3
ILMN_1720604	ARSG	0.020704046	up	NM_014960.2
ILMN_1693218	ART3	3.64E-04	up	NM_001179.3
ILMN_1800935	ARV1	0.012885517	up	NM_022786.1
ILMN_1813075	ASCL4	0.004924482	up	NM_203436.1
ILMN_1744118	ASTN2	0.007538473	up	NM_198186.2
ILMN_1748271	ATF2	0.007270547	up	NM_001880.2
ILMN_1791346	ATF3	0.012333311	up	NM_001040619.1
ILMN_1661428	ATP11C	0.03010207	up	NM_173694.3
ILMN_1696568	ATP2C1	7.28E-05	up	NM_014382.2
ILMN_1704550	AZIN1	0.023775792	up	NM_015878.4
ILMN_1687751	BAALC	2.99E-04	up	NM_001024372.1
ILMN_1772312	BARX1	1.19E-07	up	NM_021570.3
ILMN_1701170	BARX2	0.001144041	up	NM_003658.4
ILMN_1732154	BCAN	0.002338145	up	NM_021948.3
ILMN_1701165	BCL7C	0.018838897	up	XM_940124.1
ILMN_1751276	BDNF	7.62E-04	up	NM_001709.3
ILMN_1804798	BEXL1	0.020922948	up	XM_936467.2
ILMN_1699585	BHLHB5	0.009821321	up	NM_152414.3
ILMN_1708131	BHLHB9	0.015948687	up	NM_030639.1
ILMN_1679782	BLOC1S2	0.002704523	up	NM_001001342.1
ILMN_1789095	BMPR2	0.021710705	up	NM_001204.5
ILMN_1656373	BNC2	1.37E-05	up	NM_017637.5
ILMN_1712718	BPNT1	0.037641574	up	NM_006085.4
ILMN_1760246	BSN	0.001203873	up	NM_003458.3
ILMN_1704760	BZW1	0.03834131	up	NM_014670.2
ILMN_1777591	C10orf65	8.38E-04	up	NM_138413.2

ILMN_1742473	C10orf72	0.007362718	up	NM_001031746.2
ILMN_1665207	C10orf85	0.011622016	up	NM_001012711.2
ILMN_1767642	C11orf46	0.03370851	up	NM_152316.1
ILMN_1680659	C11orf63	0.009542768	up	NM_024806.2
ILMN_1704238	C14orf126	0.031055763	up	NM_080664.2
ILMN_1796377	C14orf37	0.00119423	up	NM_001001872.2
ILMN_1806456	C14orf45	1.47E-04	up	NM_025057.1
ILMN_1686835	C17orf41	0.033929706	up	XM_001134002.1
ILMN_1787280	C1orf135	0.005791685	up	NM_024037.1
ILMN_1786470	C1orf74	0.010120126	up	NM_152485.2
ILMN_1729288	C1QTNF6	0.017974203	up	NM_031910.3
ILMN_1662935	C1QTNF7	0.008259958	up	NM_031911.3
ILMN_1780356	C20orf132	1.61E-04	up	NM_152503.3
ILMN_1772869	C20orf82	0.003318641	up	NM_080826.1
ILMN_1749424	C21orf122	0.040180787	up	XM_926239.1
ILMN_1690703	C21orf34	5.79E-04	up	NM_001005734.1
ILMN_1702226	C21orf34	2.37E-05	up	NM_001005732.1
ILMN_1698166	C21orf91	0.003144156	up	NM_017447.3
ILMN_1810759	C2orf25	0.037989415	up	NM_015702.1
ILMN_1787370	C2orf56	0.014409469	up	NM_144736.4
ILMN_1671116	C3orf21	0.001698897	up	NM_152531.3
ILMN_1687084	C3orf64	0.024317747	up	NM_173654.1
ILMN_1689176	C4orf31	3.88E-05	up	NM_024574.3
ILMN_1680738	C5orf13	0.001014988	up	NM_004772.1
ILMN_1776788	C5orf41	0.027800275	up	NM_153607.1
ILMN_1658439	C5orf44	0.01904581	up	NR_003545.1
ILMN_1673478	C5orf5	0.042652655	up	NM_016603.1
ILMN_1763011	C7orf10	6.02E-05	up	NM_024728.1
ILMN_1713322	C7orf28B	0.015988192	up	NM_198097.2
ILMN_1687213	C8orf13	0.009936106	up	NM_053279.1
ILMN_1677385	C8orf40	0.02708332	up	NM_138436.2
ILMN_1713835	C9orf165	4.77E-04	up	NM_198573.2
ILMN_1674629	C9orf3	0.022964811	up	NM_032823.3
ILMN_1780460	C9orf41	0.032285925	up	NM_152420.1
ILMN_1666742	C9orf72	0.033216216	up	NM_018325.1
ILMN_1703016	CACNA1G	0.025940064	up	NM_198396.1
ILMN_1711049	CACNA2D2	0.025229456	up	NM_006030.2
ILMN_1653878	CACNB2	4.85E-07	up	NM_201597.2
ILMN_1664679	CADM2	0.025204467	up	NM_153184.2
ILMN_1705477	CAMK1D	0.039746787	up	NM_020397.2
ILMN_1714599	CAMLG	0.006898348	up	NM_001745.2
ILMN_1811957	CAMSAP1	0.004800154	up	NM_015447.1
ILMN_1685022	CAPN7	0.030020436	up	NM_014296.2
ILMN_1708983	CASC1	0.0061013	up	NM_001082972.1
ILMN_1654773	CASK	0.028394876	up	NM_003688.1
ILMN_1796762	CCDC102A	0.022284638	up	NM_033212.2
ILMN_1792456	CCDC104	0.03871413	up	NM_080667.4

ILMN_1706935	CCDC136	2.18E-04	up	NM_022742.3
ILMN_1671295	CCDC3	0.001563279	up	NM_031455.2
ILMN_1745946	CCDC5	0.00191686	up	NM_138443.2
ILMN_1731107	CCDC92	0.01937754	up	NM_025140.1
ILMN_1760088	CCKBR	0.002183554	up	NM_176875.2
ILMN_1784352	CCM2	0.010231012	up	NM_001029835.1
ILMN_1786125	CCNA2	0.019324716	up	NM_001237.2
ILMN_1752394	CCNB1IP1	0.01108644	up	NM_182849.1
ILMN_1680364	CD109	0.00724584	up	NM_133493.2
ILMN_1739164	CDC14A	0.03476669	up	NM_033312.2
ILMN_1672611	CDH11	1.11E-06	up	NM_001797.2
ILMN_1709269	CDH12	4.88E-06	up	NM_004061.2
ILMN_1748883	CDKN2D	0.018976977	up	NM_079421.2
ILMN_1753931	CDO1	0.031042693	up	NM_001801.2
ILMN_1693221	CENPH	0.02928762	up	NM_022909.3
ILMN_1742779	CENPL	6.40E-04	up	NM_033319.1
ILMN_1734176	CGA	9.56E-04	up	NM_000735.2
ILMN_1730229	CGNL1	0.005549827	up	NM_032866.3
ILMN_1674231	CHAF1B	0.005040789	up	NM_005441.2
ILMN_1744138	CHCHD7	0.028427891	up	NM_001011671.1
ILMN_1669410	CHGA	4.26E-04	up	NM_001275.3
ILMN_1677719	CHST1	0.04273998	up	NM_003654.3
ILMN_1723847	CILP	8.32E-06	up	NM_003613.2
ILMN_1716336	CKAP5	0.007886523	up	NM_001008938.1
ILMN_1729639	CLDN20	0.019205613	up	NM_001001346.2
ILMN_1723058	CLIP1	0.030072227	up	NM_002956.2
ILMN_1749071	CNIH3	0.00892887	up	NM_152495.1
ILMN_1794324	CNOT4	0.040806986	up	NM_001008225.1
ILMN_1771148	CNTN4	1.00E-04	up	NM_175612.1
ILMN_1775103	CNTNAP3B	0.024123115	up	XM_932960.1
ILMN_1711514	COCH	1.10E-08	up	NM_004086.1
ILMN_1789507	COL11A1	1.87E-08	up	NM_001854.3
ILMN_1701308	COL1A1	0.013856709	up	NM_000088.3
ILMN_1788377	COL27A1	0.017624961	up	NM_032888.2
ILMN_1729117	COL5A2	0.006407497	up	NM_000393.3
ILMN_1674050	COL8A2	5.80E-04	up	NM_005202.1
ILMN_1765644	COMMD8	0.026221633	up	NM_017845.3
ILMN_1677636	COMP	2.64E-09	up	NM_000095.2
ILMN_1666280	COX11	0.02232117	up	NM_004375.2
ILMN_1792748	CPS1	0.040711027	up	NM_001875.2
ILMN_1658040	CRABP1	5.05E-05	up	NM_004378.1
ILMN_1694432	CRIP2	0.009054358	up	NM_001312.2
ILMN_1720484	CRTAP	0.018729454	up	NM_006371.3
ILMN_1672389	CRYZ	0.014626714	up	NM_001889.2
ILMN_1762275	CSE1L	0.028114835	up	NM_177436.1
ILMN_1666894	CSPG4	2.71E-04	up	NM_001897.4
ILMN_1653129	CSTF2	0.04885106	up	NM_001325.2

ILMN_1665655	CTDSPL2	0.036727767	up	NM_016396.1
ILMN_1721901	CTNNAL1	0.002121342	up	NM_003798.2
ILMN_1757350	CTNNB1	0.036993243	up	NM_001098209.1
ILMN_1716875	CTNND2	7.47E-05	up	NM_001332.2
ILMN_1744912	CTTN	0.017896647	up	NM_005231.2
ILMN_1759766	CTXN1	0.0303406	up	NM_206833.2
ILMN_1691535	CUL4B	0.013485309	up	NM_001079872.1
ILMN_1730630	CXorf56	0.024054715	up	NM_022101.2
ILMN_1677200	CYFIP2	0.009801378	up	NM_014376.2
ILMN_1693338	CYP1B1	2.28E-04	up	NM_000104.2
ILMN_1704985	CYP27A1	0.007190268	up	NM_000784.2
ILMN_1657749	CYP27C1	0.010530582	up	NM_001001665.2
ILMN_1713918	CYTH3	9.35E-04	up	NM_004227.3
ILMN_1752668	DAAM2	0.034630317	up	NM_015345.2
ILMN_1666882	DACH1	0.005956506	up	NM_080759.3
ILMN_1755741	DACH1	4.54E-04	up	NM_080760.3
ILMN_1687556	DAPL1	9.73E-04	up	NM_001017920.1
ILMN_1810719	DCUN1D1	0.02598707	up	NM_020640.2
ILMN_1707123	DDX19B	0.049262136	up	NM_001014451.1
ILMN_1727315	DENND1A	0.008707128	up	NM_024820.2
ILMN_1802819	DEPDC1	0.038161073	up	NM_017779.3
ILMN_1670145	DFNA5	0.03330145	up	NM_004403.2
ILMN_1653047	DHX40	0.011407632	up	NM_024612.3
ILMN_1732226	DHX57	0.007022812	up	NM_144995.2
ILMN_1695226	DIAPH3	0.043876957	up	NM_030932.3
ILMN_1737650	DIO2	2.45E-08	up	NM_001007023.2
ILMN_1799796	DIS3	0.033950504	up	NM_014953.2
ILMN_1802518	DKFZp667M2411	0.030049708	up	NM_207323.1
ILMN_1815673	DKK3	4.72E-06	up	NM_015881.5
ILMN_1749829	DLG7	0.042923823	up	NM_014750.3
ILMN_1672094	DLX1	1.10E-05	up	NM_178120.4
ILMN_1812545	DMC1	0.002413563	up	NM_007068.2
ILMN_1761812	DMD	0.001503695	up	NM_004019.1
ILMN_1799516	DNAJC9	0.002346943	up	NM_015190.3
ILMN_1774261	DOK4	0.024354285	up	NM_018110.2
ILMN_1672042	DOLPP1	0.029433236	up	NM_020438.3
ILMN_1670779	DPEP1	1.25E-06	up	NM_004413.2
ILMN_1725072	DPY19L3	0.018588262	up	NM_207325.1
ILMN_1679262	DPYSL3	9.78E-05	up	NM_001387.2
ILMN_1792356	DPYSL4	3.72E-04	up	NM_006426.1
ILMN_1709257	DSCR6	0.006781379	up	NM_018962.1
ILMN_1740861	DTWD1	0.04555988	up	NM_020234.4
ILMN_1759175	DUSP10	0.005254603	up	NM_007207.3
ILMN_1697317	DYNLRB2	0.009244712	up	NM_130897.1
ILMN_1685431	DZIP1	0.010391125	up	NM_198968.2
ILMN_1653143	ECD	0.02689156	up	NM_007265.1
ILMN_1761820	EDARADD	0.007213213	up	NM_145861.2

ILMN_1796629	EDNRA	8.47E-06	up	NM_001957.1
ILMN_1703852	EFNB2	0.007764454	up	NM_004093.2
ILMN_1695606	EFNB3	5.32E-04	up	NM_001406.3
ILMN_1743199	EGR2	0.028638525	up	NM_000399.2
ILMN_1796146	EIF4E3	0.012179217	up	NM_173359.3
ILMN_1771264	ELL3	0.014355416	up	NM_025165.2
ILMN_1747499	EMID1	0.001039671	up	NM_133455.2
ILMN_1700888	ENPP1	1.11E-06	up	NM_006208.1
ILMN_1775931	EPHA3	0.03247132	up	NM_005233.3
ILMN_1715013	ERMAP	0.04247479	up	NM_001017922.1
ILMN_1720285	ESD	0.003722415	up	NM_001984.1
ILMN_1739222	ETV5	0.033626314	up	NM_004454.1
ILMN_1721713	EXOSC9	0.03424402	up	NM_001034194.1
ILMN_1652913	EZH2	0.01199195	up	NM_004456.3
ILMN_1742866	F2R	0.049956735	up	NM_001992.2
ILMN_1728923	F2RL2	0.008946881	up	NM_004101.2
ILMN_1723268	FAIM	0.005661927	up	NM_001033030.1
ILMN_1803855	FAIM2	0.031133113	up	NM_012306.2
ILMN_1714418	FAM101B	3.75E-04	up	NM_182705.2
ILMN_1723846	FAM119B	0.012831972	up	NM_015433.2
ILMN_1662949	FAM132A	0.048777208	up	NM_001014980.1
ILMN_1789407	FAM18B2	0.005155026	up	NM_145301.1
ILMN_1670352	FAM41C	0.01912746	up	XM_496333.2
ILMN_1697998	FAM54A	0.030385708	up	NM_001099286.1
ILMN_1772973	FAM82B	0.04824049	up	XM_936333.1
ILMN_1777322	FAM91A1	0.037810594	up	NM_144963.2
ILMN_1670899	FBN2	0.00129863	up	NM_001999.3
ILMN_1719755	FBN3	5.65E-04	up	NM_032447.3
ILMN_1678404	FBXO11	0.006804629	up	NM_018693.2
ILMN_1701869	FBXO22	0.019052673	up	NM_147188.1
ILMN_1749641	FBXO3	0.030551964	up	NM_012175.3
ILMN_1700232	FBXO30	2.05E-04	up	NM_032145.4
ILMN_1686697	FBXO38	0.04443192	up	NM_205836.1
ILMN_1755834	FEN1	0.027443606	up	NM_004111.4
ILMN_1789618	FER	0.015012731	up	NM_005246.2
ILMN_1671777	FGF13	0.012344884	up	NM_004114.2
ILMN_1751495	FGF14	2.83E-06	up	NM_175929.1
ILMN_1693483	FGF18	0.003768017	up	NM_003862.1
ILMN_1717737	FKBP7	0.00980497	up	NM_181342.1
ILMN_1656149	FLJ22531	0.020502899	up	XM_945494.1
ILMN_1734653	FNDC1	1.30E-05	up	NM_032532.2
ILMN_1746494	FNTA	0.037905052	up	NM_002027.2
ILMN_1704418	FOXD1	0.019953575	up	NM_004472.2
ILMN_1703174	FREM2	8.62E-04	up	NM_207361.4
ILMN_1772605	FRS3	0.017467733	up	NM_006653.3
ILMN_1798467	FRYL	0.007519289	up	NM_015030.1
ILMN_1716246	FRZB	8.74E-04	up	NM_001463.2

ILMN_1768812	FXYD6	0.004559144	up	NM_022003.1
ILMN_1703408	FZD3	0.016862461	up	NM_017412.2
ILMN_1729368	FZD8	0.03997666	up	NM_031866.1
ILMN_1681984	GALNT10	0.03762116	up	NM_017540.3
ILMN_1671221	GAPVD1	0.02218581	up	NM_015635.2
ILMN_1804569	GAS2	6.28E-06	up	NM_005256.2
ILMN_1730575	GCLC	1.11E-04	up	NM_001498.2
ILMN_1788547	GCLM	0.010215676	up	NM_002061.2
ILMN_1678191	GDF10	0.005066503	up	NM_004962.2
ILMN_1681754	GGH	0.03680292	up	NM_003878.1
ILMN_1727087	GJA1	2.74E-06	up	NM_000165.3
ILMN_1876266	GJA3	0.002039788	up	NM_021954.3
ILMN_1704376	GLDN	0.02689296	up	NM_181789.2
ILMN_1779989	GLI1	0.004283023	up	NM_005269.1
ILMN_1727577	GLI2	1.49E-04	up	NM_005270.3
ILMN_1802654	GLT8D2	0.001472977	up	NM_031302.3
ILMN_1670532	GMCL1	0.014209634	up	NM_178439.3
ILMN_1804357	GNG4	0.026303904	up	NM_001098721.1
ILMN_1691567	GNPDA2	0.00335955	up	NM_138335.1
ILMN_1738821	GOLGA2	0.04631693	up	NM_004486.4
ILMN_1795344	GOLPH4	0.00877223	up	NM_014498.2
ILMN_1688886	GPC5	0.029737432	up	NM_004466.3
ILMN_1684653	GPR115	0.016594049	up	NM_153838.3
ILMN_1787648	GPR125	0.045094553	up	XM_944791.1
ILMN_1741224	GPR137C	0.04658982	up	NM_001099652.1
ILMN_1773940	GPR161	0.00325807	up	NM_007369.2
ILMN_1738712	GPR180	0.013755705	up	NM_180989.4
ILMN_1804539	GPR89B	0.04573738	up	NM_016334.3
ILMN_1696510	GPR89C	0.02192689	up	NM_001097616.1
ILMN_1781145	GPRC5D	0.004862367	up	NM_018654.1
ILMN_1726030	GPX7	0.006155437	up	NM_015696.3
ILMN_1752965	GREM1	0.001382208	up	NM_013372.5
ILMN_1777199	GRP	3.63E-05	up	NM_001012513.1
ILMN_1762741	GTDC1	0.039369892	up	NM_001006636.1
ILMN_1802690	GULP1	0.015290747	up	NM_016315.2
ILMN_1705991	GUSBL1	0.033803754	up	NR_003504.1
ILMN_1695706	H3F3B	0.04908221	up	NM_005324.3
ILMN_1729188	HAMP	0.007783739	up	NM_021175.2
ILMN_1678812	HAPLN1	2.07E-06	up	NM_001884.2
ILMN_1710136	HDHD1A	0.018725565	up	NM_012080.3
ILMN_1790530	HECTD2	0.03335662	up	NM_182765.2
ILMN_1775268	HECW2	0.028949136	up	NM_020760.1
ILMN_1714523	HEPH	0.009422952	up	NM_138737.1
ILMN_1722829	HLF	0.014767551	up	NM_002126.4
ILMN_1654268	HMGB2	0.038999133	up	NM_002129.2
ILMN_1759676	HOXC13	0.025422955	up	NM_017410.2
ILMN_1732035	HPSE2	2.12E-04	up	NM_021828.3

ILMN_1780625	HR	0.015446958	up	NM_005144.3
ILMN_1655867	HRASLS	0.013819084	up	NM_020386.2
ILMN_1808713	HSD17B2	0.026436133	up	NM_002153.1
ILMN_1687546	HSP90AA1	0.03547446	up	NM_001017963.1
ILMN_1815445	IDS	0.023269797	up	NM_006123.2
ILMN_1717165	IGBP1	0.04870126	up	NM_001551.2
ILMN_1709613	IGF1	0.003215631	up	NM_000618.2
ILMN_1725193	IGFBP2	0.028183812	up	NM_000597.2
ILMN_1679897	IGFL3	0.007302711	up	NM_207393.1
ILMN_1653459	IL11RA	0.04359791	up	NM_147162.1
ILMN_1753823	IL17D	5.40E-04	up	NM_138284.1
ILMN_1688152	IL27RA	0.04155184	up	NM_004843.2
ILMN_1707979	INCA	0.016521674	up	NM_001007232.1
ILMN_1802377	IQCG	0.013386988	up	NM_032263.2
ILMN_1662852	IQCK	0.006594126	up	NM_153208.1
ILMN_1698985	IQWD1	0.008761443	up	NM_018442.2
ILMN_1670000	IQWD1	0.038591877	up	NM_001017977.1
ILMN_1665792	ITGA2	0.023263	up	NM_002203.3
ILMN_1776516	ITPKA	0.014500871	up	NM_002220.1
ILMN_1755727	JARID1B	0.016706996	up	NM_006618.3
ILMN_1750373	KAL1	1.48E-04	up	NM_000216.2
ILMN_1722533	KATNAL1	0.026483621	up	NM_001014380.1
ILMN_1779480	KCMF1	0.044857983	up	XM_001133393.1
ILMN_1669669	KCMF1	0.017583096	up	XM_001133393.1
ILMN_1775348	KCNH8	0.003402262	up	NM_144633.2
ILMN_1744387	KCNIP1	0.003983025	up	NM_001034838.1
ILMN_1701173	KCNK6	0.04800234	up	NM_004823.1
ILMN_1713901	KDELR3	0.016855149	up	NM_016657.1
ILMN_1722820	KDELR3	0.01956081	up	NM_006855.2
ILMN_1797822	KIAA0746	0.004487359	up	NM_015187.3
ILMN_1813704	KIAA1199	1.64E-05	up	NM_018689.1
ILMN_1652371	KIAA1324L	0.001089126	up	NM_152748.2
ILMN_1785036	KIAA1345	3.82E-06	up	XM_944553.1
ILMN_1805992	KIAA1598	0.025197823	up	NM_018330.4
ILMN_1775965	KIAA1622	0.036377553	up	NM_058237.1
ILMN_1811820	KIAA1853	2.49E-05	up	NM_194286.2
ILMN_1678097	KIAA2010	0.039965227	up	NM_017936.3
ILMN_1674486	KIAA2026	0.043929726	up	NM_001017969.2
ILMN_1735552	KIF1B	0.002432569	up	NM_015074.2
ILMN_1695658	KIF20A	0.036686976	up	NM_005733.1
ILMN_1811472	KIF23	0.04548294	up	NM_004856.4
ILMN_1788160	KIF5B	0.04160765	up	NM_004521.1
ILMN_1654916	KIF7	0.048218176	up	NM_198525.1
ILMN_1654653	KLC1	0.004709879	up	NM_005552.4
ILMN_1693401	KLHL28	0.011734125	up	NM_017658.3
ILMN_1666191	KLK13	0.024380587	up	NM_015596.1
ILMN_1669700	KPNA5	0.037110936	up	NM_002269.2

ILMN_1770612	KRT15	0.03233079	up	NM_002275.2
ILMN_1704497	KRT25	3.08E-07	up	NM_181534.2
ILMN_1723404	KRT26	0.002149272	up	NM_181539.3
ILMN_1680828	KRT27	5.34E-06	up	NM_181537.3
ILMN_1666887	KRT28	9.77E-04	up	NM_181535.3
ILMN_1807249	KRT32	0.00216896	up	NM_002278.3
ILMN_1731800	KRT33A	0.04606809	up	NM_004138.2
ILMN_1770015	KRT33B	0.020894432	up	NM_002279.3
ILMN_1770228	KRT34	4.67E-04	up	NM_021013.3
ILMN_1716136	KRT71	1.17E-05	up	NM_033448.1
ILMN_1695812	KRT72	0.015709529	up	NM_080747.1
ILMN_1672120	KRT73	9.92E-04	up	NM_175068.2
ILMN_1713272	KRT74	4.45E-07	up	NM_175053.3
ILMN_1721247	KRT75	9.16E-06	up	NM_004693.2
ILMN_1801442	KRT81	0.010102137	up	NM_002281.2
ILMN_1765072	KRT85	7.32E-05	up	NM_002283.3
ILMN_1798206	KRTAP11-1	0.001761286	up	NM_175858.2
ILMN_1791951	KRTAP13-1	0.00224599	up	NM_181599.1
ILMN_1758039	KRTAP13-2	2.75E-04	up	NM_181621.2
ILMN_1707652	KRTAP1-5	0.008493112	up	NM_031957.1
ILMN_1713715	KRTAP17-1	6.47E-04	up	NM_031964.1
ILMN_1801138	KRTAP19-1	7.11E-05	up	NM_181607.1
ILMN_1763883	KRTAP19-3	2.38E-04	up	NM_181609.3
ILMN_1656051	KRTAP19-4	4.16E-05	up	NM_181610.1
ILMN_1805469	KRTAP19-5	4.90E-04	up	NM_181611.1
ILMN_1784216	KRTAP19-6	0.003439437	up	NM_181612.2
ILMN_1748852	KRTAP19-7	1.12E-04	up	NM_181614.1
ILMN_1719105	KRTAP20-1	0.002228409	up	NM_181615.1
ILMN_1740258	KRTAP20-2	0.004192657	up	NM_181616.1
ILMN_1658448	KRTAP2-1	0.006838814	up	XM_926554.2
ILMN_1806253	KRTAP2-1	0.00420819	up	XM_926554.2
ILMN_1676146	KRTAP2-2	0.015488961	up	XM_001127069.1
ILMN_1780344	KRTAP2-2	0.001638977	up	XM_001127069.1
ILMN_1908490	KRTAP24-1	0.009727919	up	NM_001085455.1
ILMN_1786908	KRTAP26-1	0.006388697	up	NM_203405.1
ILMN_1652439	KRTAP3-1	0.013196624	up	NM_031958.1
ILMN_1662081	KRTAP3-2	0.008925332	up	NM_031959.2
ILMN_1662497	KRTAP3-2	0.001878311	up	NM_031959.2
ILMN_1780326	KRTAP4-1	0.00722498	up	NM_033060.2
ILMN_1698584	KRTAP4-12	0.009562717	up	NM_031854.2
ILMN_1746434	KRTAP4-3	0.006180455	up	NM_033187.1
ILMN_1714010	KRTAP4-4	0.004468684	up	NM_032524.1
ILMN_1659475	KRTAP4-5	0.00584365	up	NM_033188.3
ILMN_1680432	KRTAP4-7	0.03343505	up	NM_033061.2
ILMN_1718202	KRTAP4-7	2.16E-04	up	NM_033061.2
ILMN_1684885	KRTAP4-8	0.03156877	up	XM_001130340.1
ILMN_1802360	KRTAP5-2	0.00733924	up	NM_001004325.1

ILMN_1744383	KRTAP5-4	0.048858836	up	NM_001012709.1
ILMN_1791777	KRTAP5-7	0.046599075	up	NM_001012503.1
ILMN_1728440	KRTAP6-3	0.037387002	up	NM_181605.3
ILMN_1730562	KRTAP7-1	0.002481221	up	XM_927228.2
ILMN_1694419	KRTAP8-1	0.001731926	up	NM_175857.3
ILMN_1693976	KRTAP9-3	5.22E-04	up	NM_031962.2
ILMN_1696738	KRTAP9-4	0.012323748	up	NM_033191.2
ILMN_1715753	KRTAP9-5	0.015430899	up	XM_926671.2
ILMN_1716822	KRTAP9-8	0.008272229	up	NM_031963.2
ILMN_1652175	KRTAP9-8	0.01848548	up	NM_031963.2
ILMN_1710314	KRTAP9-8	0.008578285	up	XM_937208.1
ILMN_1653824	LAMC2	0.026530892	up	NM_005562.1
ILMN_1688642	LAMC3	9.23E-05	up	NM_006059.3
ILMN_1679185	LEF1	0.0049165	up	NM_016269.2
ILMN_1781256	LEFTY2	1.62E-05	up	NM_003240.2
ILMN_1702310	LGR5	7.95E-06	up	NM_003667.2
ILMN_1807016	LHX2	4.46E-06	up	NM_004789.3
ILMN_1792712	LOC201725	0.02858789	up	NM_001008393.1
ILMN_1710954	LOC283932	6.61E-04	up	NM_175901.3
ILMN_1675803	LOC285550	0.012433608	up	XR_017764.1
ILMN_1795835	LOC338758	0.009278894	up	XM_931359.2
ILMN_1716382	LOC387882	0.003820722	up	NM_207376.1
ILMN_1787931	LOC389517	0.03910316	up	NR_003664.1
ILMN_1725528	LOC400657	0.014962669	up	NM_001008234.1
ILMN_1769288	LOC402560	0.033962637	up	XM_944321.1
ILMN_1669657	LOC440345	0.020472817	up	XM_933717.1
ILMN_1710192	LOC440345	0.030708369	up	XM_933707.1
ILMN_1761422	LOC440349	0.021338342	up	XM_496129.2
ILMN_1719579	LOC441081	0.030826017	up	XR_017029.1
ILMN_1749304	LOC643389	0.032260675	up	XM_926721.1
ILMN_1813801	LOC643668	0.028617267	up	XR_016986.1
ILMN_1658304	LOC643669	3.03E-04	up	XM_933621.2
ILMN_1673980	LOC643669	0.001055041	up	XM_933621.2
ILMN_1671800	LOC643911	0.015495298	up	XM_931911.2
ILMN_1709583	LOC644019	0.009576981	up	XM_933103.1
ILMN_1769024	LOC644113	0.001346269	up	XM_927336.1
ILMN_1676719	LOC644330	0.043337945	up	XR_017492.1
ILMN_1694878	LOC644979	0.013560504	up	XM_928051.1
ILMN_1699058	LOC644979	0.001859498	up	XM_928051.1
ILMN_1661647	LOC646144	0.008471328	up	XM_935296.1
ILMN_1667813	LOC648000	0.021683343	up	XM_371757.5
ILMN_1687058	LOC648494	0.03710689	up	XM_937544.1
ILMN_1694767	LOC652688	0.041085716	up	XM_942297.1
ILMN_1777299	LOC652771	0.027499229	up	XM_942413.1
ILMN_1722032	LOC653060	0.042337514	up	XM_925779.1
ILMN_1708627	LOC653226	0.021772292	up	XM_927451.2
ILMN_1763404	LOC653226	0.039778255	up	XM_927451.2

ILMN_1772207	LOC653377	0.047418542	up	XM_929420.1
ILMN_1755808	LOC654194	0.03222181	up	XM_942669.1
ILMN_1692072	LOC728006	0.017720504	up	XM_001128698.1
ILMN_1770498	LOC728411	0.006940598	up	XM_001128030.1
ILMN_1722223	LOC728452	0.008196823	up	XM_001130826.1
ILMN_1790819	LOC728556	0.002714157	up	XM_001129672.1
ILMN_1658563	LOC728760	0.037865892	up	XM_001133055.1
ILMN_1760665	LOC728765	0.003183094	up	XM_001133057.1
ILMN_1776838	LOC728895	0.037615236	up	XR_015405.1
ILMN_1657962	LOC728946	0.002006371	up	XM_001128870.1
ILMN_1669119	LOC728946	0.001748054	up	XM_001128870.1
ILMN_1776354	LOC728951	0.01039726	up	XM_001128885.1
ILMN_1711628	LOC728951	0.004924798	up	XM_001128885.1
ILMN_1681558	LOC728956	0.014949477	up	XM_001128901.1
ILMN_1710220	LOC729985	0.02767012	up	XM_001131964.1
ILMN_1658586	LOC730012	0.005063967	up	XM_001132077.1
ILMN_1666522	LOC730077	0.03801388	up	XM_001132307.1
ILMN_1680774	LOC730994	0.019036321	up	XM_001132373.1
ILMN_1753980	LOC731950	0.04569231	up	XR_016039.1
ILMN_1723232	LOC96610	0.045235123	up	NM_080926.1
ILMN_1695880	LOX	0.014997173	up	NM_002317.3
ILMN_1734950	LOXL1	0.037837457	up	NM_005576.2
ILMN_1692623	LPHN3	0.001665032	up	NM_015236.3
ILMN_1711807	LPHN3	8.34E-06	up	NM_015236.3
ILMN_1677765	LRP8	0.00216752	up	NM_017522.3
ILMN_1709717	LRRC15	0.001880884	up	NM_130830.2
ILMN_1688479	LRRC42	0.045386985	up	NM_052940.3
ILMN_1720511	LRRN1	1.61E-04	up	NM_020873.5
ILMN_1773650	LRRN3	5.43E-04	up	NM_001099660.1
ILMN_1665219	LTBP4	0.03615184	up	NM_001042545.1
ILMN_1790529	LUM	5.67E-04	up	NM_002345.3
ILMN_1696295	LY6G6D	1.16E-04	up	NM_021246.2
ILMN_1708081	LYCAT	0.04550711	up	NM_001002257.1
ILMN_1691606	LYG2	2.31E-04	up	NM_175735.3
ILMN_1776724	LYPD6	0.001018045	up	NM_194317.2
ILMN_1815705	LZTFL1	0.012237945	up	NM_020347.2
ILMN_1798663	MAB21L2	1.16E-05	up	XM_001130278.1
ILMN_1737935	MACF1	0.03834082	up	NM_012090.3
ILMN_1777564	MAD2L1	0.004694807	up	NM_002358.2
ILMN_1775522	MAGED1	0.011346467	up	NM_001005332.1
ILMN_1722022	MAGEE1	0.001315174	up	NM_020932.1
ILMN_1775853	MAGI1	0.018132199	up	NM_001033057.1
ILMN_1723020	MAP3K1	0.026623048	up	NM_005921.1
ILMN_1807042	MARCKS	0.03397949	up	NM_002356.5
ILMN_1704236	MAX	0.026580477	up	NM_145112.1
ILMN_1663195	MCM7	0.03596516	up	NM_182776.1
ILMN_1705685	MEIS1	0.022536743	up	NM_002398.2

ILMN_1785795	METAP1	0.034661744	up	NM_015143.1
ILMN_1670420	METAP2	0.011622725	up	NM_006838.3
ILMN_1691570	METTL5	0.033588223	up	NM_014168.2
ILMN_1787981	MFAP2	0.001515591	up	NM_017459.1
ILMN_1811779	MGC24103	1.77E-05	up	XR_001080.1
ILMN_1737283	MGC39900	5.87E-04	up	NM_194324.1
ILMN_1731640	MGC39900	1.57E-06	up	NM_194324.1
ILMN_1740512	MGC39900	1.88E-06	up	XM_936687.1
ILMN_1681780	MKX	1.83E-04	up	NM_173576.1
ILMN_1746277	MLLT4	0.005734644	up	NM_005936.2
ILMN_1733937	MMD	0.029295221	up	NM_012329.2
ILMN_1732197	MN1	5.86E-04	up	NM_002430.2
ILMN_1761788	MOXD1	0.004860421	up	NM_015529.2
ILMN_1687501	MOXD1	0.009373017	up	NM_015529.2
ILMN_1679995	MPP6	0.013412334	up	NM_016447.2
ILMN_1766154	MRPL30	0.016280143	up	NM_145212.2
ILMN_1713088	MSI2	0.001935349	up	NM_138962.2
ILMN_1713156	MSL3L1	0.026162012	up	NM_078629.1
ILMN_1660222	MTBP	0.04666706	up	NM_022045.3
ILMN_1814230	MTCP1	9.40E-05	up	NM_014221.3
ILMN_1672884	MTFMT	0.011577568	up	NM_139242.2
ILMN_1787658	MTMR7	1.43E-04	up	NM_004686.3
ILMN_1776352	MUTED	0.03023373	up	NM_201280.1
ILMN_1756541	MXD4	0.03680593	up	NM_006454.2
ILMN_1803213	MXRA5	2.19E-06	up	NM_015419.2
ILMN_1811972	MYCBP2	0.029294595	up	NM_015057.3
ILMN_1653761	MYCN	1.96E-04	up	NM_005378.4
ILMN_1815154	MYH10	0.00146824	up	NM_005964.1
ILMN_1684255	MYL4	0.004688924	up	NM_002476.2
ILMN_1713450	MYL6B	6.37E-04	up	NM_002475.3
ILMN_1774350	MYOZ3	0.008195959	up	NM_133371.2
ILMN_1699208	NAP1L1	0.034385514	up	NM_139207.1
ILMN_1785406	NAP1L3	0.016980657	up	NM_004538.3
ILMN_1705346	NBEA	0.049874384	up	NM_015678.3
ILMN_1698067	NBPF14	0.04739732	up	NM_015383.1
ILMN_1680348	NBPF3	0.043127574	up	NM_032264.2
ILMN_1724718	NCK2	0.005648808	up	NM_001004720.1
ILMN_1769040	NCOA2	0.017885288	up	NM_006540.2
ILMN_1664511	NDC80	0.037233874	up	NM_006101.1
ILMN_1725417	NELL2	0.006382547	up	NM_006159.1
ILMN_1790909	NFE2L2	0.02658531	up	NM_006164.2
ILMN_1804610	NFX1	0.03867265	up	NM_147134.1
ILMN_1739521	NLGN1	0.029693574	up	NM_014932.2
ILMN_1789094	NLP	0.025351744	up	NM_025176.4
ILMN_1786197	NR2F1	1.17E-05	up	NM_005654.4
ILMN_1680673	NT5DC1	0.049686387	up	NM_152729.2
ILMN_1717765	NUDT11	0.043832537	up	NM_018159.3

ILMN_1787885	NUDT18	0.041367278	up	NM_024815.3
ILMN_1706378	NUDT4	0.033823203	up	NM_019094.3
ILMN_1768293	NUP155	0.023747092	up	NM_153485.1
ILMN_1762674	NUP43	0.008527799	up	NM_198887.1
ILMN_1726720	NUSAP1	0.03789374	up	NM_018454.5
ILMN_1746116	OCA2	0.008919477	up	NM_000275.1
ILMN_1700306	OCIAD2	0.009000907	up	NM_001014446.1
ILMN_1772286	OCIAD2	9.98E-04	up	NM_152398.2
ILMN_1749846	OMD	0.047537606	up	NM_005014.1
ILMN_1789096	OSTalpha	3.55E-05	up	NM_152672.4
ILMN_1772667	P2RY1	0.040629435	up	NM_002563.2
ILMN_1758888	PADI3	1.92E-05	up	NM_016233.2
ILMN_1721770	PAPPA	0.018405296	up	NM_002581.3
ILMN_1659470	PBRM1	0.04314143	up	NM_181042.2
ILMN_1688500	PCDH10	1.55E-05	up	NM_032961.1
ILMN_1703572	PCDH20	3.68E-05	up	NM_022843.2
ILMN_1710544	PCDH7	0.027688595	up	NM_002589.2
ILMN_1675428	PCDHGC3	0.037201047	up	NM_032402.1
ILMN_1807994	PCNP	0.017121052	up	NM_020357.1
ILMN_1755582	PCSK1N	1.18E-07	up	NM_013271.2
ILMN_1767934	PCSK5	5.32E-05	up	NM_006200.2
ILMN_1668850	PCSK9	0.016041154	up	NM_174936.2
ILMN_1775163	PDE1A	7.62E-05	up	NM_001003683.1
ILMN_1778924	PDE1A	0.002161799	up	NM_001003683.1
ILMN_1653793	PDPK1	0.017928034	up	NM_002613.3
ILMN_1675695	PDS5B	0.021780716	up	NM_015032.1
ILMN_1742949	PDZRN3	0.002058431	up	XM_001133042.1
ILMN_1780132	PELI2	0.014875079	up	NM_021255.2
ILMN_1726711	PENK	0.03578862	up	NM_006211.2
ILMN_1737416	PGBD3	0.006007375	up	NM_170753.1
ILMN_1790533	PHACTR2	0.032659344	up	NM_001100164.1
ILMN_1812552	PHCA	0.026773587	up	NM_018367.4
ILMN_1699496	PHF21A	0.0057238	up	NM_016621.2
ILMN_1682181	PHKG1	0.036900006	up	NM_006213.2
ILMN_1769406	PIAS2	0.022703683	up	NM_004671.2
ILMN_1694799	PIAS2	0.035710648	up	NM_004671.2
ILMN_1778709	PICALM	0.017907958	up	NM_007166.2
ILMN_1741017	PIP4K2B	0.013629039	up	NM_003559.4
ILMN_1780671	PLEKHG3	0.025160683	up	NM_015549.1
ILMN_1753312	PLXDC2	8.45E-04	up	NM_032812.7
ILMN_1734276	PMEPA1	0.004617266	up	NM_199169.1
ILMN_1790778	PNMA2	0.001174455	up	NM_007257.4
ILMN_1783805	PNMA3	0.005980583	up	NM_013364.4
ILMN_1751773	POLD3	0.049330216	up	NM_006591.1
ILMN_1658221	POLH	0.034619704	up	NM_006502.1
ILMN_1657317	POLR2J	0.021713272	up	NM_006234.4
ILMN_1795678	POLR3C	0.028159443	up	NM_006468.6

ILMN_1675406	PPAPDC1B	0.02363948	up	NM_032483.2
ILMN_1791093	PPHLN1	0.024911059	up	NM_016488.5
ILMN_1808333	PPP1R7	0.037565555	up	NM_002712.1
ILMN_1796962	PPP3R1	0.00584256	up	NM_000945.3
ILMN_1655077	PRDM1	0.039877392	up	NM_001198.2
ILMN_1683148	PRICKLE2	9.41E-04	up	NM_198859.2
ILMN_1742922	PRIM2A	0.032828745	up	XM_001134299.1
ILMN_1782403	PRR11	0.001246469	up	NM_018304.2
ILMN_1779778	PRR19	0.012252023	up	NM_199285.2
ILMN_1781791	PRRG1	0.006758875	up	NM_000950.1
ILMN_1811851	PRRX1	0.006974135	up	NM_006902.3
ILMN_1739496	PRRX1	2.95E-06	up	NM_006902.3
ILMN_1797776	PRSS23	0.009052384	up	NM_007173.4
ILMN_1769580	PRSS35	0.025630075	up	NM_153362.1
ILMN_1717477	PSD3	0.015753385	up	NM_015310.3
ILMN_1808548	PSEN1	6.04E-04	up	NM_007318.1
ILMN_1671843	PSRC1	0.037999783	up	NM_001032290.1
ILMN_1800331	PTCH1	0.00222237	up	NM_001083605.1
ILMN_1717393	PTCHD1	0.030257396	up	NM_173495.2
ILMN_1785699	PTHLH	2.10E-04	up	NM_198964.1
ILMN_1795166	PTHR1	0.003334388	up	NM_000316.2
ILMN_1743049	PWP1	0.027573034	up	NM_007062.1
ILMN_1810486	RAB34	0.024833001	up	NM_031934.3
ILMN_1803197	RAB3IP	0.018956816	up	NM_175624.2
ILMN_1680712	RAET1E	0.027618779	up	NM_139165.1
ILMN_1659836	RAET1G	0.010079304	up	NM_001001788.2
ILMN_1798855	RASSF9	0.010119026	up	NM_005447.3
ILMN_1804737	RAVER2	0.024729019	up	NM_018211.2
ILMN_1665040	RBMS3	0.015024101	up	NM_001003793.1
ILMN_1656837	RBP1	0.001552086	up	NM_002899.2
ILMN_1708537	RBPJ	0.042133674	up	NM_203284.1
ILMN_1662129	RCN2	0.002013989	up	NM_002902.1
ILMN_1666057	REEP2	0.003716297	up	NM_016606.2
ILMN_1719811	REM1	0.006373521	up	NM_014012.4
ILMN_1746359	RERG	0.048378445	up	NM_032918.1
ILMN_1658143	RFC3	0.007625598	up	NM_002915.3
ILMN_1659364	RFC5	0.03724203	up	NM_007370.3
ILMN_1756102	RFX3	0.033571523	up	NM_134428.1
ILMN_1669983	RGS22	0.047838967	up	NM_015668.2
ILMN_1758067	RGS4	1.43E-06	up	NM_005613.3
ILMN_1651554	RGS5	0.041940566	up	NM_003617.2
ILMN_1716019	RHBDL3	1.90E-05	up	NM_138328.2
ILMN_1741281	RNF175	0.004688924	up	NM_173662.2
ILMN_1710758	RNF20	0.044933785	up	NM_019592.5
ILMN_1768139	RNU12	0.020350123	up	NR_000041.1
ILMN_1746658	RORB	0.004281427	up	NM_006914.3
ILMN_1798880	RPA4	0.038094472	up	NM_013347.2

ILMN_1800796	RPL37	3.14E-04	up	NM_000997.3
ILMN_1712413	RPL39L	0.021143893	up	NM_052969.1
ILMN_1665425	RPRM	0.015371566	up	NM_019845.2
ILMN_1771593	RRM1	0.049258985	up	NM_001033.2
ILMN_1678669	RRM2	0.010998067	up	NM_001034.1
ILMN_1788223	RSPH3	0.034821257	up	NM_031924.3
ILMN_1721657	RSU1	0.026383905	up	NM_012425.3
ILMN_1712545	S100A3	5.15E-05	up	NM_002960.1
ILMN_1709067	SAMD11	3.00E-04	up	NM_152486.2
ILMN_1755215	SAMD5	1.93E-04	up	NM_001030060.1
ILMN_1752793	SAP18	0.039745186	up	NM_005870.3
ILMN_1728298	SBK1	0.014287277	up	NM_001024401.2
ILMN_1655405	SCARF2	0.001839404	up	NM_153334.3
ILMN_1813561	SCIN	0.001538372	up	NM_033128.1
ILMN_1714738	SCMH1	0.006183812	up	NM_012236.2
ILMN_1653861	SCMH1	0.012544329	up	NM_001031694.1
ILMN_1684211	SEC14L2	0.030321931	up	NM_012429.1
ILMN_1656927	SEMA5A	0.001385267	up	NM_003966.1
ILMN_1880012	SEMA5A	0.00992426	up	NM_003966.2
ILMN_1713529	SEMA6A	0.016884087	up	NM_020796.3
ILMN_1673369	SEPHS1	0.012963989	up	NM_012247.3
ILMN_1788874	SERPINA3	0.012831721	up	NM_001085.4
ILMN_1655595	SERPINE2	0.03007203	up	NM_006216.2
ILMN_1730794	SERTAD4	0.004958937	up	NM_019605.2
ILMN_1720513	SETBP1	0.015933627	up	NM_015559.1
ILMN_1722898	SFRP2	2.60E-07	up	NM_003013.2
ILMN_1741007	SGCA	0.003065019	up	NM_000023.1
ILMN_1702835	SH3BGRL	0.043470517	up	NM_003022.1
ILMN_1760990	SH3GL3	5.04E-04	up	NM_003027.2
ILMN_1760412	SHISA2	1.18E-05	up	NM_001007538.1
ILMN_1782938	SLC16A10	0.008612189	up	NM_018593.3
ILMN_1729691	SLC16A6	0.008492753	up	NM_004694.3
ILMN_1732410	SLC16A9	3.92E-05	up	NM_194298.1
ILMN_1656904	SLC1A4	2.59E-04	up	NM_003038.2
ILMN_1763609	SLC22A16	4.42E-07	up	NM_033125.2
ILMN_1720311	SLC25A46	7.76E-04	up	NM_138773.1
ILMN_1732371	SLC26A10	0.017829161	up	NM_001018084.1
ILMN_1656129	SLC39A10	0.002622232	up	NM_020342.1
ILMN_1758673	SLC44A1	0.040082984	up	NM_080546.3
ILMN_1700695	SLC44A1	0.011047328	up	NM_080546.3
ILMN_1693912	SLC47A2	2.03E-06	up	NM_152908.3
ILMN_1683694	SLC6A4	0.001682807	up	NM_001045.3
ILMN_1673586	SLC6A6	0.049261298	up	NM_003043.3
ILMN_1781400	SLC7A2	6.23E-04	up	NM_001008539.2
ILMN_1807894	SLC7A8	0.032160603	up	NM_182728.1
ILMN_1678928	SLCO3A1	0.03808165	up	NM_013272.2
ILMN_1736969	SLITRK3	1.02E-04	up	NM_014926.2

ILMN_1778595	SLN	3.87E-04	up	NM_003063.2
ILMN_1685369	SLU7	0.048880678	up	NM_006425.4
ILMN_1674551	SMAD5	0.03180293	up	NM_005903.5
ILMN_1767068	SMAD6	0.024531608	up	NM_005585.3
ILMN_1815385	SMAD9	0.005226682	up	NM_005905.3
ILMN_1791702	SMARCA2	0.034709603	up	NM_003070.3
ILMN_1740555	SNAP25	1.99E-04	up	NM_130811.1
ILMN_1807969	SNCAIP	1.80E-05	up	NM_005460.2
ILMN_1743217	SNORD32A	0.003107423	up	NR_000021.1
ILMN_1667609	SNORD35A	0.009579973	up	NR_000018.1
ILMN_1720794	SNORD4B	0.044690922	up	NR_000009.1
ILMN_1680393	SNORD55	0.034346696	up	NR_000015.2
ILMN_1753241	SNTA1	0.020513035	up	NM_003098.2
ILMN_1666502	SOBP	0.014968948	up	NM_018013.3
ILMN_1785286	SOCS5	0.016493376	up	NM_144949.2
ILMN_1668463	SON	0.023735164	up	NM_058183.2
ILMN_1773459	SOX11	0.048643015	up	NM_003108.3
ILMN_1783185	SOX21	0.044019707	up	NM_007084.2
ILMN_1815745	SOX4	0.035712145	up	NM_003107.2
ILMN_1728256	SPAG9	0.019023644	up	NM_003971.3
ILMN_1796734	SPARC	0.040983185	up	NM_003118.2
ILMN_1796738	SPAST	0.02737191	up	NM_014946.3
ILMN_1791890	SPON1	0.001390091	up	NM_006108.2
ILMN_1691884	STC2	0.005210242	up	NM_003714.2
ILMN_1745593	STMN1	0.003150567	up	NM_005563.3
ILMN_1664014	STOX1	0.00644399	up	NM_152709.3
ILMN_1684402	STXBP5	0.049068324	up	NM_139244.2
ILMN_1686981	SULF2	4.54E-05	up	NM_018837.2
ILMN_1667460	SULF2	0.00180342	up	NM_018837.2
ILMN_1702009	SV2A	0.041620836	up	NM_014849.2
ILMN_1806306	SV2B	0.025702443	up	NM_014848.3
ILMN_1671404	SVIL	0.03590897	up	NM_003174.3
ILMN_1785175	SWAP70	0.007497082	up	NM_015055.2
ILMN_1771261	SYNC1	2.65E-04	up	NM_030786.1
ILMN_1707326	TASP1	0.029598065	up	NM_017714.2
ILMN_1708147	TBPL1	0.008611809	up	NM_004865.2
ILMN_1698015	TBX1	0.007488569	up	NM_080647.1
ILMN_1765310	TCEAL2	0.002463285	up	NM_080390.3
ILMN_1656399	TCEAL8	0.00226645	up	NM_001006684.1
ILMN_1669832	TCF12	6.61E-04	up	NM_207038.1
ILMN_1681248	TCHH	1.46E-06	up	NM_007113.2
ILMN_1675808	TCHHL1	0.002949683	up	NM_001008536.1
ILMN_1758404	TFAP2B	0.006373322	up	NM_003221.3
ILMN_1661717	TFDP1	6.60E-04	up	NM_007111.3
ILMN_1685567	TGDS	0.025646081	up	NM_014305.2
ILMN_1687652	TGFB3	1.41E-04	up	NM_003239.1
ILMN_1713873	THSD7B	0.045045972	up	NM_001080427.1

ILMN_1654609	TIGA1	0.017670758	up	NM_053000.1
ILMN_1793829	TMCO1	0.04607351	up	NM_019026.2
ILMN_1730645	TMEFF2	0.002323186	up	NM_016192.2
ILMN_1789112	TMEM145	0.004254278	up	NM_173633.2
ILMN_1785191	TMEM14A	0.008029615	up	NM_014051.3
ILMN_1792455	TMEM158	0.003212411	up	NM_015444.2
ILMN_1758679	TMEM168	0.034203816	up	NM_022484.4
ILMN_1699121	TMEM16B	3.49E-05	up	NM_020373.1
ILMN_1771120	TMEM45B	0.009764248	up	NM_138788.3
ILMN_1689704	TMEM5	0.035448555	up	NM_014254.1
ILMN_1779182	TMEM98	0.014915553	up	NM_001033504.1
ILMN_1725014	TMPRSS6	9.15E-04	up	NM_153609.2
ILMN_1681737	TMSL8	4.81E-04	up	NM_021992.2
ILMN_1719759	TNC	0.029986117	up	NM_002160.2
ILMN_1724096	TNK1	0.028162204	up	NM_003985.1
ILMN_1763587	TNMD	5.97E-06	up	NM_022144.1
ILMN_1753789	TNN	5.33E-06	up	NM_022093.1
ILMN_1786347	TNPO1	0.023030235	up	NM_153188.2
ILMN_1752409	TNRC6A	0.010344627	up	NM_014494.2
ILMN_1746871	TNRC6C	0.037033487	up	NM_018996.2
ILMN_1802642	TOM1L1	0.03270722	up	NM_005486.1
ILMN_1788886	TOX	0.046016295	up	NM_014729.2
ILMN_1780349	TPR	4.10E-04	up	NM_003292.2
ILMN_1737146	TRAM1	0.024805164	up	NM_014294.4
ILMN_1754130	TRIM52	0.024562078	up	NM_032765.2
ILMN_1656910	TRIM6	0.038148817	up	NM_058166.3
ILMN_1732722	TRNAU1AP	0.04831879	up	NR_003109.1
ILMN_1691648	TRO	0.04005805	up	NM_177556.1
ILMN_1744574	TRUB1	0.011544467	up	NM_139169.4
ILMN_1743933	TSHZ3	3.56E-04	up	NM_020856.2
ILMN_1710899	TSPAN18	0.003458023	up	NM_130783.3
ILMN_1699980	TSPAN18	0.017148878	up	NM_130783.3
ILMN_1737972	TSPYL5	0.042936493	up	NM_033512.2
ILMN_1710303	TTC25	0.024315692	up	NM_031421.2
ILMN_1755487	TTC28	0.01618132	up	XM_929318.2
ILMN_1670385	TUBAL3	1.20E-04	up	NM_024803.1
ILMN_1774689	TUSC3	0.014038974	up	NM_006765.2
ILMN_1726967	TWSG1	0.017168526	up	NM_020648.3
ILMN_1680314	TXN	0.001281734	up	NM_003329.2
ILMN_1714730	UBE2C	0.032969892	up	NM_181803.1
ILMN_1683817	UBE2Q2	0.03839948	up	NM_173469.1
ILMN_1731412	UBTD2	0.009429049	up	NM_152277.2
ILMN_1757387	UCHL1	0.008534522	up	NM_004181.3
ILMN_1757646	UFM1	0.018573945	up	NM_016617.1
ILMN_1808677	UGT2B17	0.001459401	up	NM_001077.2
ILMN_1755897	UGT2B7	0.00164272	up	XM_001128725.1
ILMN_1679194	UGT2B7	2.95E-04	up	XM_001128725.1

ILMN_1786065	UHRF1	0.015553951	up	NM_001048201.1
ILMN_1774860	UTP23	0.017218975	up	NM_032334.1
ILMN_1690524	VAMP7	0.033104874	up	NM_005638.3
ILMN_1685929	VANGL1	0.025370462	up	NM_138959.2
ILMN_1796216	VASH1	7.04E-05	up	NM_014909.3
ILMN_1794638	VIP	0.010128088	up	NM_194435.1
ILMN_1730740	VSIG8	0.001764873	up	NM_001013661.1
ILMN_1716674	VWA2	0.021133773	up	NM_198496.1
ILMN_1736448	VWC2	0.002129684	up	NM_198570.2
ILMN_1700248	WDR86	0.009303755	up	NM_198285.1
ILMN_1662065	WDR92	0.03367645	up	NM_138458.2
ILMN_1669114	WNK4	1.29E-04	up	NM_032387.3
ILMN_1800317	WNT5A	0.002035804	up	NM_003392.3
ILMN_1781222	XGPY2	0.006588111	up	NR_003254.1
ILMN_1734316	YME1L1	0.014668033	up	NM_139312.1
ILMN_1711069	YPEL5	0.001359791	up	NM_016061.1
ILMN_1666111	YTHDC1	2.88E-04	up	NM_001031732.2
ILMN_1674385	YWHAQ	1.63E-04	up	NM_006826.2
ILMN_1698803	ZAK	0.04767509	up	NM_016653.2
ILMN_1768110	ZAK	0.04922983	up	NM_133646.2
ILMN_1737320	ZDHHC15	0.001675167	up	NM_144969.1
ILMN_1657606	ZFHX4	0.007717819	up	NM_024721.3
ILMN_1772522	ZFP161	0.032300986	up	NM_003409.2
ILMN_1656413	ZMPSTE24	0.016728459	up	NM_005857.3
ILMN_1713521	ZNF205	0.023698468	up	NM_003456.2
ILMN_1670895	ZNF207	6.76E-04	up	NM_001032293.2
ILMN_1676983	ZNF300	0.048612647	up	NM_052860.1
ILMN_1662021	ZNF326	0.017773768	up	NM_182976.1
ILMN_1662340	ZNF358	0.033839773	up	NM_018083.4
ILMN_1658094	ZNF365	0.017210664	up	NM_014951.2
ILMN_1732909	ZNF483	0.029291637	up	NM_001007169.1
ILMN_1787265	ZNF503	0.025829142	up	NM_032772.3
ILMN_1726199	ZNF594	0.033474397	up	NM_032530.1
ILMN_1781720	ZNF720	0.03894867	up	NM_001004300.1
ILMN_1669696	ZNF792	0.03878799	up	NM_175872.4
ILMN_1819784		4.60E-05	up	DR979451
ILMN_1819854		0.001003504	up	CR624627
ILMN_1823128		0.008900551	up	AA406061
ILMN_1823231		0.001123996	up	CR603183
ILMN_1825368		1.75E-05	up	BF515803
ILMN_1828197		0.048143838	up	CD511953
ILMN_1828967		0.023703134	up	AK022085
ILMN_1831943		0.018413315	up	CX869259
ILMN_1835686		0.003654624	up	AL831861
ILMN_1836208		0.018747749	up	BP421183
ILMN_1847308		0.04154459	up	D87470
ILMN_1849186		0.042524174	up	BC004287

ILMN_1851547	0.016090212	up	AK125664
ILMN_1852122	0.001776064	up	U92981
ILMN_1853711	0.031008063	up	CD511973
ILMN_1859036	0.017261466	up	AL832853
ILMN_1861376	0.012221037	up	AL080095
ILMN_1862001	0.047604	up	BU145923
ILMN_1863509	0.019614512	up	AK094703
ILMN_1866954	0.049856756	up	AJ420536
ILMN_1869934	0.01000539	up	BX100997
ILMN_1870111	0.0378307	up	AK123449
ILMN_1873374	0.016700681	up	AL355741
ILMN_1874575	1.47E-04	up	CB133377
ILMN_1877022	0.021724354	up	BF510052
ILMN_1877990	0.006318758	up	AV702201
ILMN_1878019	0.002912375	up	AL512695
ILMN_1880457	0.001151441	up	AA450153
ILMN_1890773	0.004575201	up	CB135276
ILMN_1902493	0.015190728	up	CD104817
ILMN_1905356	0.04630368	up	BU147750
ILMN_1905821	0.007902303	up	BQ707492
ILMN_1906110	0.005196081	up	CR610854
ILMN_1907915	0.034167375	up	BP398298
ILMN_1909542	0.039539885	up	CB957775
ILMN_1910572	0.030868297	up	AV722509
