# Supplementary material for: Transcriptome Analysis of Skin Photoaging in Chinese Females Reveals the Involvement of Skin Homeostasis and Metabolic Changes
Source: PLoS One. 2013 Apr 24;8(4):e61946. doi: 10.1371/journal.pone.0061946 (PMC3634825; doi:10.1371/journal.pone.0061946)
Supplement: Table S2 — List of the top 100 shared DEGs identified by both GeneSpring and GSEA. (PDF) [file pone.0061946.s003.pdf]

Symbol	GeneSpring P-value	GSEA score	Regulation
BARX1	1.19E-07	1.004	up
BNC2	1.37E-05	0.799	up
C21orf34	1.47E-04	0.76	up
CDH11	1.11E-06	0.888	up
CDH12	4.88E-06	0.822	up
CILP	8.32E-06	0.795	up
COCH	1.10E-08	1.138	up
COL11A1	1.87E-08	1.143	up
COMP	2.64E-09	1.188	up
DKK3	4.72E-06	0.82	up
DLX1	1.10E-05	0.86	up
DPEP1	1.25E-06	0.923	up
EDNRA	8.47E-06	0.82	up
ENPP1	1.11E-06	0.886	up
FNDC1	1.30E-05	0.776	up
GJA1	2.74E-06	0.844	up
GRP	3.63E-05	0.72	up
HAPLN1	2.07E-06	0.959	up
KIAA1199	1.64E-05	0.76	up
KIAA1345	3.82E-06	0.828	up
KIAA1853	2.49E-05	0.743	up
KRT25	3.08E-07	0.946	up
KRT27	5.34E-06	0.81	up
KRT71	1.17E-05	0.772	up
KRT74	4.45E-07	0.93	up
KRT75	9.16E-06	0.786	up
LEFTY2	1.62E-05	0.758	up
LGR5	7.95E-06	0.851	up
LHX2	4.46E-06	0.831	up
LPHN3	8.34E-06	0.793	up
MAB21L2	1.16E-05	0.946	up
MGC24103	1.77E-05	0.762	up
MXRA5	2.19E-06	0.854	up
NR2F1	1.17E-05	0.795	up
OSTalpha	3.55E-05	0.73	up
PADI3	1.92E-05	0.756	up
PCDH10	1.55E-05	0.894	up
PCDH20	3.68E-05	0.72	up
PCSK1N	1.18E-07	1.012	up
RGS4	1.43E-06	1.008	up
RHBDL3	1.90E-05	0.771	up
SFRP2	2.60E-07	0.967	up
SHISA2	1.18E-05	0.778	up
SLC22A16	4.42E-07	0.936	up
SLC47A2	2.03E-06	0.871	up
SNCAIP	1.80E-05	0.792	up

TCHH	1.46E-06	0.872	up
TMEM16B	3.49E-05	0.731	up
TNMD	5.97E-06	0.812	up
TNN	5.33E-06	0.847	up
CDH4	9.20E-07	-0.967	down
CLEC10A	7.17E-05	-0.684	down
SCGB1D2	5.34E-05	-0.701	down
IQGAP2	1.05E-06	-0.891	down
ALDH1L1	3.88E-05	-0.714	down
EDAR	1.06E-04	-0.667	down
PTPRT	5.10E-06	-0.816	down
ADH1B	2.80E-05	-0.75	down
RPTN	4.85E-07	-0.957	down
TDRD10	9.52E-05	-0.692	down
CYP4B1	1.31E-05	-0.767	down
DKK1	1.40E-04	-0.667	down
FUT2	4.29E-05	-0.725	down
GALE	1.87E-05	-0.749	down
GCGR	9.57E-05	-0.694	down
RSPO1	7.75E-06	-0.794	down
SCARA5	3.55E-05	-0.721	down
HOXA2	7.45E-08	-1.071	down
HOXA5	4.02E-07	-0.948	down
HOXB2	5.38E-07	-0.92	down
HOXB3	5.54E-06	-0.813	down
HOXB5	1.22E-05	-0.834	down
HOXC4	4.70E-05	-0.704	down
HOXC8	6.71E-07	-0.911	down
IL1A	1.06E-04	-0.672	down
SCGB2A1	6.51E-05	-0.693	down
ATP6V1B1	1.49E-04	-0.647	down
GDPD2	6.02E-06	-0.804	down
PPP1R3D	9.99E-05	-0.671	down
KIAA1467	2.46E-06	-0.849	down
CEACAM1	1.51E-04	-0.654	down
GREM2	3.08E-06	-0.849	down
CA2	1.05E-04	-0.667	down
SKAP2	2.87E-05	-0.742	down
COL23A1	1.04E-05	-0.778	down
C3orf72	1.82E-05	-0.751	down
FLJ35880	8.74E-05	-0.674	down
LOC440731	2.69E-05	-0.732	down
NAP5	7.29E-05	-0.683	down
SRD5A2L2	2.13E-05	-0.745	down
UBR4	6.39E-06	-0.801	down
ADH1A	1.63E-04	-0.699	down
TSHZ2	1.63E-04	-0.648	down

SEC23B	1.64E-04	-0.642	down
FOXA1	1.71E-04	-0.64	down
KCNS1	1.71E-04	-0.652	down
TBX3	1.71E-04	-0.64	down
VSIG4	1.80E-04	-0.638	down
SLC4A11	1.83E-04	-0.64	down
PI15	1.92E-04	-0.637	down
